# Substituted cysteine accessibility method (SCAM) analysis of the transport domain of human concentrative nucleoside transporter 3 (hCNT3) and other family members reveals features of structural and functional importance

**DOI:** 10.1074/jbc.M116.743997

**Published:** 2017-04-06

**Authors:** Ras Mulinta, Sylvia Y. M. Yao, Amy M. L. Ng, Carol E. Cass, James D. Young

**Affiliations:** From the Membrane Protein Disease Research Group, Departments of ‡ Physiology and; § Oncology, University of Alberta, Edmonton, Alberta T6G 2H7, Canada and; the ¶ Cross Cancer Institute, Edmonton, Alberta T6G 2H7, Canada

**Keywords:** membrane protein, membrane transport, nucleoside/nucleotide transport, recombinant protein expression, transporter, nucleoside transporter, human concentrative nucleoside transporter 3 (hCNT3), nucleoside transport, substituted cysteine accessibility (SCAM) analysis

## Abstract

The human SLC28 family of concentrative nucleoside transporter (CNT) proteins has three members: hCNT1, hCNT2, and hCNT3. Na^+^-coupled hCNT1 and hCNT2 transport pyrimidine and purine nucleosides, respectively, whereas hCNT3 transports both pyrimidine and purine nucleosides utilizing Na^+^ and/or H^+^ electrochemical gradients. *Escherichia coli* CNT family member NupC resembles hCNT1 in permeant selectivity but is H^+^-coupled. Using heterologous expression in *Xenopus* oocytes and the engineered cysteine-less hCNT3 protein hCNT3(C−), substituted cysteine accessibility method analysis with the membrane-impermeant thiol reactive reagent *p*-chloromercuribenzene sulfonate was performed on the transport domain (interfacial helix 2, hairpin 1, putative transmembrane domain (TM) 7, and TM8), as well as TM9 of the scaffold domain of the protein. This systematic scan of the entire C-terminal half of hCNT3(C−) together with parallel studies of the transport domain of wild-type hCNT1 and the corresponding TMs of cysteine-less NupC(C−) yielded results that validate the newly developed structural homology model of CNT membrane architecture for human CNTs, revealed extended conformationally mobile regions within transport-domain TMs, identified pore-lining residues of functional importance, and provided evidence of an emerging novel elevator-type mechanism of transporter function.

## Introduction

Specialized nucleoside transporter proteins are required for passage of nucleosides and hydrophilic nucleoside analogs across biological membranes. Physiologic nucleosides serve as central salvage metabolites in nucleotide biosynthesis, and nucleoside analogs are used in chemotherapy of cancer and antiviral diseases (1, 2). Adenosine modulates numerous cellular events via purino-receptor cell signaling pathways, including neurotransmission, vascular tone, immune responses, and other physiological processes (3, 4).

Human nucleoside transporter proteins are divided into two families: the SLC29 equilibrative nucleoside transporter (ENT)^2^ family and the SLC28 concentrative nucleoside transporter (CNT) family (3, 5–7). hENTs mediate bidirectional fluxes of purine and pyrimidine nucleosides down their concentration gradients and are ubiquitously found in most, possibly all, cell types (8). hENT1/2 also transport nucleobases (9, 10). hCNTs are inwardly directed Na^+^-dependent nucleoside transporters found predominantly in intestinal and renal epithelial and other specialized cell types (11, 12). hCNT1 and hCNT2 are pyrimidine and purine nucleoside selective, respectively, and couple Na^+^:nucleoside cotransport with 1:1 stoichiometry (13–19). hCNT3 transports both pyrimidine and purine nucleosides and couples Na^+^:nucleoside cotransport with 2:1 stoichiometry (11, 19, 20). hCNT3 is also capable of H^+^:nucleoside cotransport with a coupling stoichiometry of 1:1, whereby one of the two Na^+^-binding sites also functionally interacts with H^+^ (19, 20).

**Figure 1.**
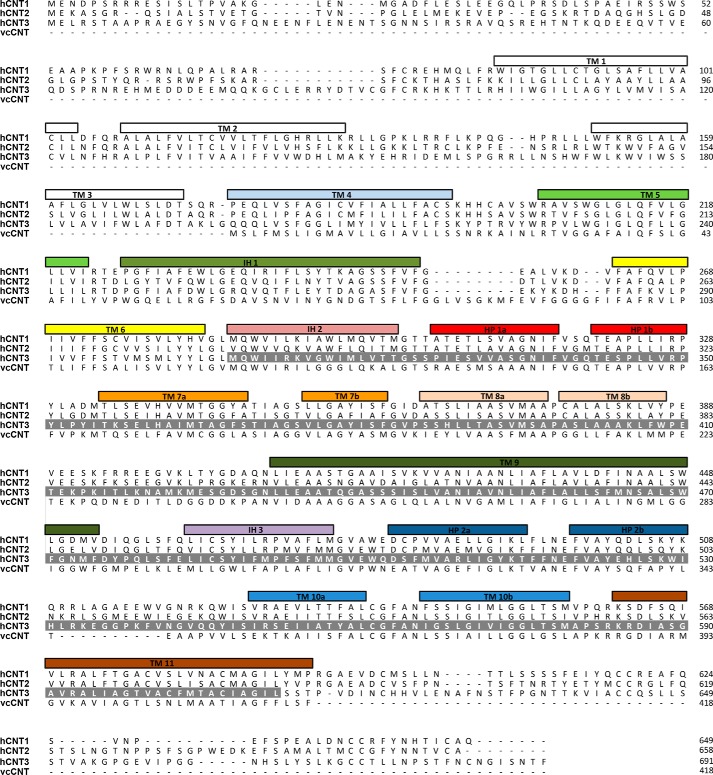
**hCNT3 topology.** Shown is the sequence alignment of hCNT1 (U62968), hCNT2 (NP_004203.2), hCNT3 (AF305210), and vcCNT (NP_231982.1). *Bars* representing helices have the same color scheme as in Fig. 2. Residues studied by SCAM analysis in the present study and Refs. 28 and 29 are highlighted in *gray*.

**Figure 2.**
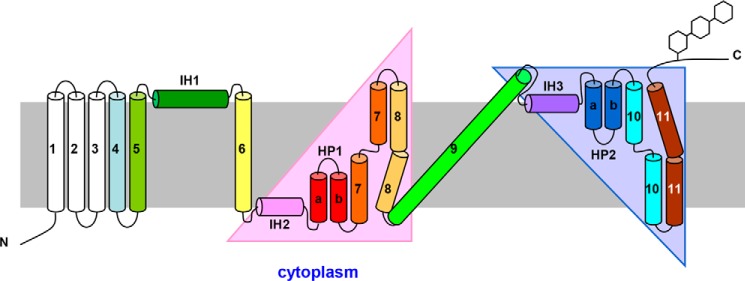
**Predicted topology of human nucleoside transporter hCNT3 based upon that of its counterpart from *V. cholerae* (21).** The N-terminal transmembrane helices designated TM1, TM2, and TM3 and the C-terminal extramembranous tail with *N*-glycosylation sites are not present in the bacterial protein.

Recently, the structure of the bacterial Na^+^-linked CNT from *Vibrio cholerae* (vcCNT) with bound uridine and sodium was determined at a resolution of 2.4 Å (21). The *Vibrio* CNT is homotrimeric in membrane architecture and shows 39% amino acid sequence identity with hCNT3 (Fig. 1). Each promoter contains eight transmembrane helices (TM1–TM8), three interfacial helices (IH1–IH3) oriented parallel to the plane of the membrane, and two re-entrant helical hairpins (HP1 and HP2) that have opposite orientations in the membrane. Both termini are periplasmic (21). The predicted membrane topology of homologous hCNT3 has extended extracellular C-terminal regions containing multiple sites of *N*-linked glycosylation, with an additional three TMs at its N terminus that are not essential for transport activity (22) (Fig. 2).

By analogy with the bacterial structure, hCNT3 topology can be subdivided into an outer or “scaffold” domain comprising TM4, TM5, IH1, TM6, and TM9, which surrounds an inner or “transport” domain that can itself be divided into two structural subdomains that are related by an internal 2-fold pseudo-symmetry. The first subdomain comprises IH2, HP1, TM7, and TM8, whereas the second subdomain comprises IH3, HP2, TM10, and TM11 (Fig. 2). hCNT3 residues potentially contributing to uridine binding are from both subdomains, in particular at the tips of HP1 and HP2 and in the unwound regions of TM7 and TM10. The bacterial crystal structure also predicts a hCNT3 sodium-binding site located between the tip of HP1 and the unwound portion of TM7.

Chimeric studies involving hCNTs and hfCNT, a CNT from the ancient marine prevertebrate the Pacific hagfish *Eptatretus stouti*, have revealed that the functional domains responsible for CNT nucleoside selectivity and cation coupling reside within the C-terminal HP1-TM11 half of the protein (20, 23). NupC, a H^+^-coupled CNT family member from *Escherichia coli*, lacks TMs 1–3 but otherwise shares a topology similar to that of its eukaryote counterparts (24, 25).

A functional cysteine-less version of hCNT3 has been generated by mutagenesis of endogenous cysteine residues to serine, resulting in the cysteine-less construct hCNT3(C−). Employed originally in a yeast expression system for substituted cysteine accessibility method (SCAM) analysis of what is now recognized as the second or C terminus transport domain using methanethiosulfonate reagents (26), we have also characterized hCNT3(C−) in the *Xenopus* oocyte expression system (27) and have undertaken SCAM analysis of the same region (IH3, HP2, TM10, and TM11) with the alternative thiol-specific reagent *p*-chloromercuribenzene sulfonate (PCMBS) (28, 29). Measured by transport inhibition, reactivity of introduced cysteine residues with PCMBS, which is both membrane-impermeant and hydrophilic, indicates pore-lining status and access from the extracellular medium; the ability of a permeant to protect against this inhibition denotes location within, or closely adjacent to, the permeant-binding pocket (30, 31).

Building upon prior work with methanethiosulfonate reagents and other structure/function studies of hCNTs (29, 32, 33), our PCMBS SCAM analysis of the hCNT3(C−) C-terminal transport subdomain (IH3, HP2, TM10, and TM11) identified important residues of functional importance (28, 29). These include a cluster of conformationally responsive residue positions in TM10 (Ile^554^, Tyr^558^, and Cys^561^) that exhibit H^+^-activated inhibition by PCMBS, with uridine protection evident for Tyr^558^ and Cys^561^ (29). Glu^519^ in the HP2 region of hCNT3 and the corresponding residue in hCNT1 (Glu^498^) were also identified as having key roles in permeant and cation coupling/translocation (28, 32, 33), hCNT3 E519C being centrally positioned within the highly conserved motif (G/A)*X*K*X*_3_NEFVA(Y/M/F) (33).

To complete investigation of the transport domain of hCNT3, the present study reports a comprehensive PCMBS SCAM analysis of the corresponding N-terminal transport subdomain of this transporter (IH2, HP1, TM7, and TM8), as well as the long linker TM9 region of the protein. Together with parallel studies of HP1 and TM7 of Na^+^-specific wild-type hCNT1 and the corresponding TMs of H^+^-specific cysteine-less NupC(C−), our results validate the newly predicted structural model of CNT membrane architecture, reveal extended conformationally mobile regions at the tip of HP1 and within TM7, identify further pore-lining residues of functional importance, and support an emerging novel elevator-type mechanism of transporter function.

## Results

All 14 endogenous cysteine residues of hCNT3 were replaced with serine to produce hCNT3(C−), a cysteine-less hCNT3 construct (26, 27). hCNT3(C−) retained wild-type hCNT3 functional activity and was used as a template for the construction of single-cysteine mutants prior to scanning for functional activity and inhibition by PCMBS. Complementing previous analyses of IH3, HP2, TM10, and TM11 (26, 28, 29), the 172 residues spanning a region between and including IH2, HP1, TM7, TM8, and TM9 that were investigated in the present study are highlighted in Fig. 1 in which the two helical portions of HP1 are identified as HP1a and HP1b, whereas those in discontinuous TM7 are correspondingly identified as TM7a and TM7b. We also undertook parallel SCAM analyses of HP1 and TM7 of wild-type hCNT1 and the two equivalent transmembrane regions of a cysteine-less version of *E. coli* NupC (NupC(C−)).

### Functional activity of single hCNT3(C−) cysteine mutants

hCNT3 transports nucleosides using both Na^+^ and H^+^ electrochemical gradients (19, 20). Therefore, to examine the functional activity of single-cysteine mutants, uptake of 10 μm radiolabeled uridine was determined in both Na^+^-containing, H^+^-reduced medium (100 mm NaCl, pH 8.5) and Na^+^-free, acidified medium (100 mm ChCl, pH 5.5, respectively). The Na^+^-containing medium was buffered at a pH of 8.5 to avoid the small but significant H^+^ activation of hCNT3 shown previously to occur at pH 7.5 (19, 20). In earlier work, we verified that Na^+^-coupled uridine transport by hCNT3 at pH 8.5 is kinetically indistinguishable from that at pH 7.5 (27). Initial rates of transport (± S.E.) for each mutant, in units of pmol/oocyte·min^−1^, are given in supplemental Table S1. These and other reported flux values represent mediated transport activities, defined as the differences in uptake between RNA transcript-injected and control water-injected oocytes, and are from representative experiments. Uptake of 10 μm radiolabeled uridine (100 mm NaCl, pH 8.5) by oocytes producing hCNT3(C−) varied in different experiments between 2 and 4 pmol/oocyte·min^−1^; the corresponding uptake of uridine by water-injected oocytes was <0.02 pmol/oocyte·min^−1^ (data not shown).

Two mutants that exhibited uridine uptake values of <0.1 pmol/oocyte·min^−1^ were excluded from further analysis (supplemental Table S1). In both cases, the mutation to cysteine resulted in a protein with low functional activity in both Na^+^-containing, H^+^-reduced and Na^+^-free, acidified media (100 mm NaCl, pH 8.5, and 100 mm ChCl, pH 5.5, respectively). The two residues were Glu^343^ in HP1b and Tyr^379^ in TM7b. Cell-surface labeling with sulfo-NHS-LC-biotin and immobilized streptavidin resin were used to distinguish cell-surface proteins from those associated with total (plasma + intracellular) membranes. Both mutants, which had electrophoretic mobilities similar to that of hCNT3(C−), were present at cell surfaces in greatly reduced amounts so that no conclusions could be reached regarding their transport activities (data not shown).

**Figure 3.**
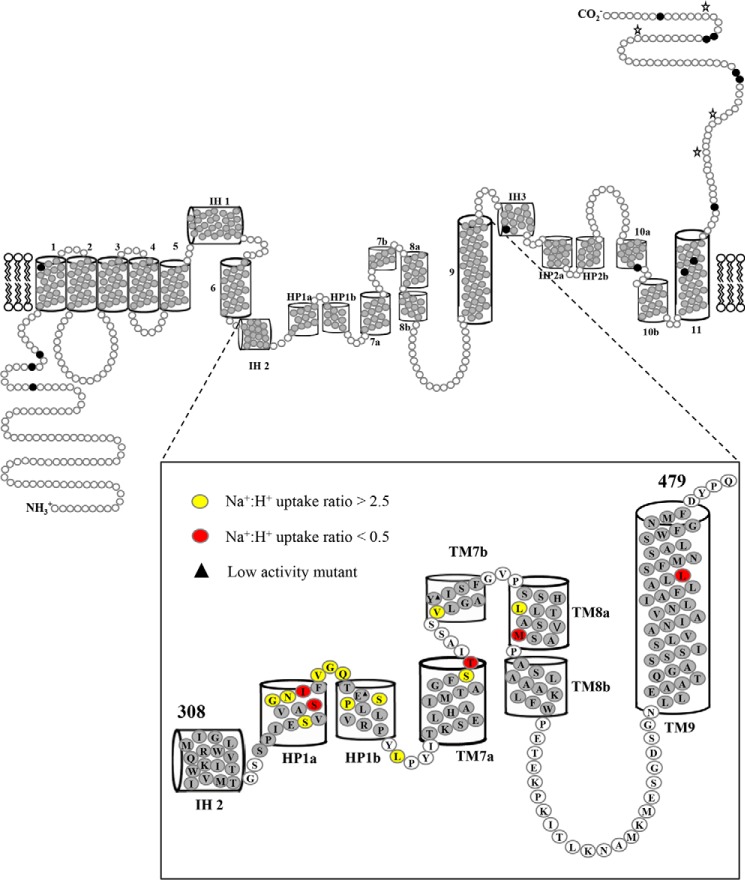
**hCNT3 residues in the IH 2-TM9 region with altered Na^+^:H^+^ uridine uptake ratios.** hCNT3(C−) mutants exhibiting Na^+^:H^+^ uridine uptake ratios of >2.5 are shown in *yellow*, and those with uptake ratios < 0.5 are shown in *red*. Low-activity mutants with uridine transport rates of <0.1 pmol/oocyte·min^−1^ in both Na^+^-containing, H^+^-reduced and Na^+^-free, acidified media (100 mm NaCl, pH 8.5, and ChCl, pH 5.5, respectively) are indicated by *black triangles*. Corresponding numerical values are given in supplemental Table S1.

To facilitate comparisons between the remaining 170 mutants, supplemental Table S1 additionally presents uridine transport activity of each construct calculated as the flux ratio of Na^+^-mediated to H^+^-mediated uptake (Na^+^:H^+^). The corresponding Na^+^:H^+^ ratios of uridine uptake (10 μm) for wild-type hCNT3 and cysteine-less hCNT3(C−) were ∼1.7 and 1.0, respectively (averaged results from multiple experiments; data not shown), and were consistent with results of previous studies (19, 20, 27, 28). Residue mutations that resulted in Na^+^:H^+^ ratios of uridine uptake of <0.5 and >2.5 (supplemental Table S1) are highlighted in the hCNT3 topology schematic shown in Fig. 3.

Most constructs exhibited Na^+^:H^+^ uptake ratios similar to those of either hCNT3 or hCNT3(C−). In contrast, there were eight mutants in HP1 that gave Na^+^:H^+^ ratios of >2.5 (S330C(C−), G335C(C−), N336C(C−), V339C(C−), G340C(C−), Q341C(C−), S344C(C−), and P345C(C−) with values of 2.7, 3.5, 6.5, 2.9, 10.2, 4.6, 9.4, and 9.2, respectively), and two mutants, S334C(C−) and I337C(C−), that exhibited low Na^+^:H^+^ ratios of 0.3 and 0.4, respectively.

One mutant in the loop between HP1 and TM7 and a cluster of three mutants in TM7 also exhibited altered cation-coupling properties: loop mutant L352C(C−) gave a Na^+^:H^+^ uptake ratio of 4.5, and within TM7, mutants S369C(C−), T370C(C−), and V375C(C−) had values of 2.9, 0.2, and 8.0, respectively. One mutant in TM8 had a Na^+^:H^+^ ratio of >2.5 (L389C(C−), with a value of 5.9, and one mutant each in TMs 8 and 9, M395C(C−) and L461C(C−), exhibited low Na^+^:H^+^ ratios of 0.38 and 0.36, respectively. All mutants in the large loop linking TMs 8 and 9 resembled hCNT3 and hCNT3(C−).

### PCMBS inhibition of single hCNT3(C−) cysteine mutants

**Figure 4.**
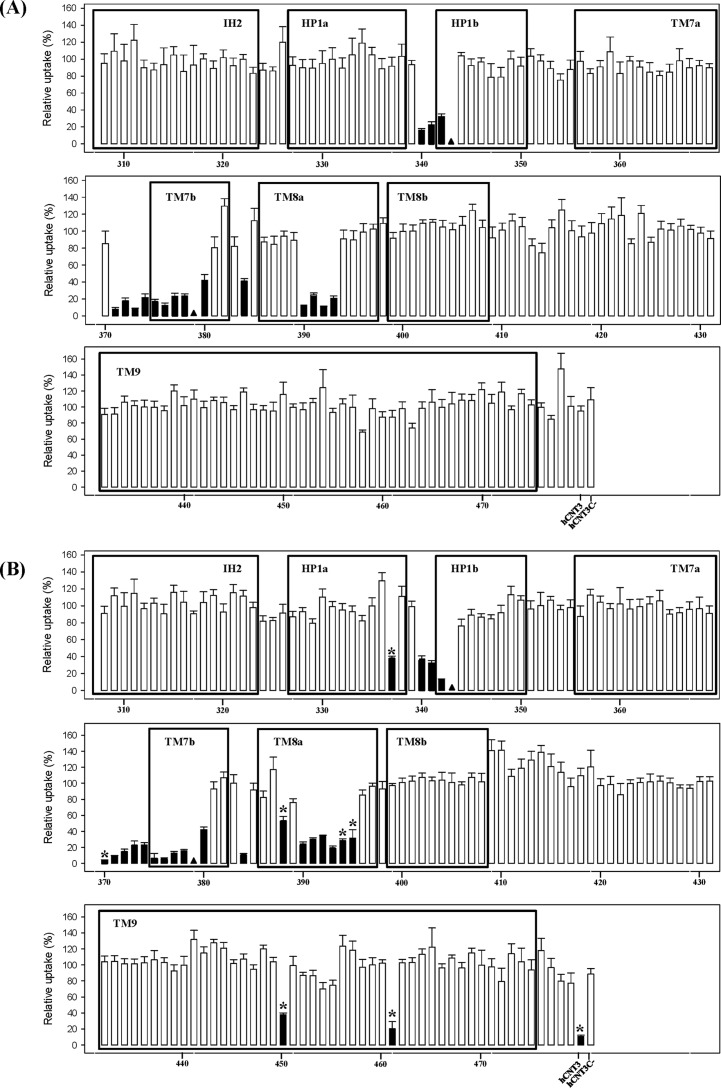
**PCMBS inhibition of residues in the IH2-TM9 region of hCNT3(C−).** Mediated influx of 10 μm radiolabeled uridine in Na^+^-containing, H^+^-reduced (*A*) or Na^+^-free, acidified (*B*) medium (100 mm NaCl, pH 8.5, or 100 mm ChCl, pH 5.5, respectively) was measured following 10 min of incubation on ice in the same medium (*A* or *B*, respectively) in the presence of 200 μm PCMBS. *Black columns* indicate residue positions inhibited by PCMBS; the *asterisk* identifies those residues that exhibited differential inhibition by PCMBS in the two media. Low-activity mutants for which inhibition was not determined are indicated by *black triangles*. The data are presented as mediated transport, calculated as uptake in RNA-injected oocytes *minus* uptake in water-injected oocytes, and normalized to influx of uridine in the absence of inhibitor. Each value is the mean ± S.E. of 10–12 oocytes. hCNT3 and hCNT3(C−) were included as controls in all experiments.

**Figure 5.**
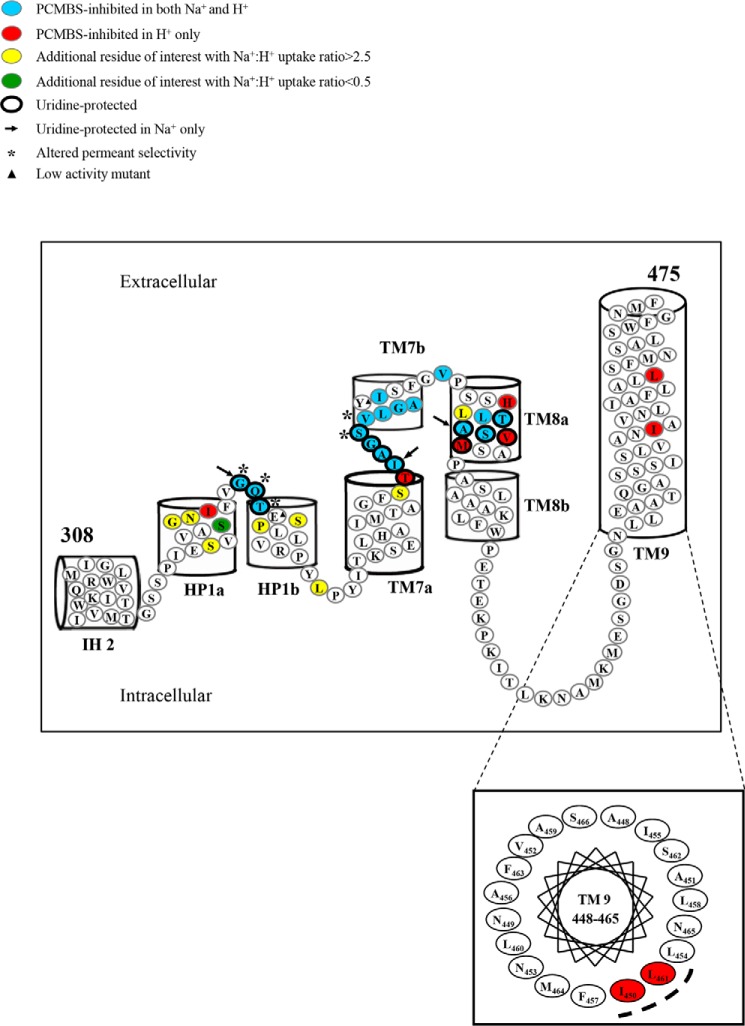
**hCNT3 IH2-TM9 region depicting PCMBS-inhibited and uridine-protected residues.** hCNT3(C−) mutants exhibiting inhibition of uridine uptake following incubation with PCMBS in both Na^+^-containing, H^+^-reduced and Na^+^-free, acidified media are indicated in *blue*, and those that were inhibited only in Na^+^-free, acidified medium are indicated in *red*. Residues protected from PCMBS inhibition by excess unlabeled uridine are *outlined* in *black*. The three residues, Gly^340^ in the connecting region between HP1a and HP1b, Ile^371^ in the connecting region between TM7a and TM7b, and Ala^392^ in TM8a, which were inhibited by PCMBS in both media but protected from that inhibition only in the presence of Na^+^-containing, H^+^-reduced medium, are indicated by *black arrows*. Additional residues of interest with Na^+^:H^+^ uridine uptake ratios of >2.5 or <0.5 but not inhibited by PCMBS are highlighted in *yellow* and *green*, respectively. Low-activity mutants are indicated by *black triangles*, and those with altered permeant selectivity are indicated by *asterisks*. The helical wheel projection for TM9 in the *inset* shows clustering of the two PCMBS-sensitive residues to one face of the helix. The corresponding numerical values are given in Table 1.

Wild-type hCNT3 has previously been reported to be sensitive to inhibition by PCMBS under acidic conditions only (*i.e.* in Na^+^-free, acidified medium), there being no PCMBS inhibition in either Na^+^-containing, H^+^-reduced or Na^+^-free, H^+^-reduced medium (29). Therefore, each of the single-cysteine mutants of hCNT3(C−) was tested for inhibition by PCMBS both in Na^+^-containing, H^+^-reduced medium (100 mm NaCl, pH 8.5) and in Na^+^-free, acidified medium (100 mm ChCl, pH 5.5). After 10-min exposures to 200 μm PCMBS, uptake of 10 μm radiolabeled uridine was assayed in medium of the same composition. Exposure to PCMBS was performed on ice to minimize its diffusion across oocyte plasma membranes (29, 34). In ascending numerical order of residue position, results for each mutant calculated as a percentage of mediated uridine uptake in the absence of PCMBS are presented in Fig. 4. For screening purposes, a residue was considered to be PCMBS-inhibitable upon exhibiting >20% inhibition of uridine uptake by PCMBS. A schematic of the locations of PCMBS-inhibitable residues is presented in Fig. 5, and the corresponding numerical flux values are presented in Table 1. Fig. 4 and Table 1 also include control data for wild-type hCNT3 (only inhibited by PCMBS in Na^+^-free, acidified medium) and hCNT3(C−) (unaffected by PCMBS either in Na^+^-containing, H^+^-reduced medium or in Na^+^-free, acidified medium).

**Table 1:** **Effects of PCMBS on uridine uptake in *Xenopus* oocytes expressing hCNT3(C−) single-cysteine mutants** Influx of 10 μm [^3^H]uridine was measured in Na^+^-containing, H^+^-reduced or Na^+^-free, acidified medium (100 mm NaCl, pH 8.5, or 100 mm ChCl, pH 5.5, respectively) following 10 min of incubation on ice in the absence or presence of 200 μm PCMBS or 200 μm PCMBS + 20 mm uridine in medium of the same composition used to determine uptake. The values are corrected for basal non-mediated uptake in control water-injected oocytes and are presented as percentages of mediated uridine influx in the absence of inhibitor for each individual mutant. Each value is the mean ± S.E. of 10–12 oocytes.

TM		Na^+^ (100 mm NaCl, pH 8.5)		H^+^ (100 mm ChCl, pH 5.5)	
		+ PCMBS*^a^*	+ PCMBS, + uridine	+ PCMBS*^a^*	+ PCMBS, + uridine
		%		%	
HP1a	I337C (C−)	73 ± 10	78 ± 7	40 ± 4	43 ± 4
HP1a-HP1b loop	G340C (C−)	16 ± 2	105 ± 9*^b^*	48 ± 5	57 ± 10
HP1a-HP1b loop	Q341C (C−)	23 ± 3	86 ± 14*^b^*	48 ± 2	83 ± 6*^b^*
HP1b	T342C (C−)	32 ± 3	76 ± 4*^b^*	18 ± 2	75 ± 6*^b^*
TM7a-TM7b loop	T370C (C−)	104 ± 12	104 ± 10	4 ± 1	64 ± 4*^b^*
TM7a-TM7b loop	I371C (C−)	7 ± 2	85 ± 10*^b^*	9 ± 1	19 ± 3
TM7a-TM7b loop	A372C (C−)	17 ± 3	100 ± 10*^b^*	30 ± 4	115 ± 17*^b^*
TM7a-TM7b loop	G373C (C−)	8 ± 1	56 ± 4*^b^*	43 ± 10	96 ± 14*^b^*
TM7a-TM7b loop	S374C (C−)	22 ± 5	89 ± 17*^b^*	42 ± 7	87 ± 10*^b^*
TM7b	V375C (C−)	16 ± 3	25 ± 3	8 ± 5	11 ± 4
TM7b	L376C (C−)	56 ± 11	60 ± 14	27 ± 2	27 ± 3
TM7b	G377C (C−)	48 ± 11	66 ± 10	33 ± 6	57 ± 10
TM7b	A378C (C−)	27 ± 11	32 ± 11	22 ± 1	23 ± 3
TM7b	I380C (C−)	42 ± 7	62 ± 10	42 ± 3	45 ± 4
TM7b-TM8a loop	V384C (C−)	19 ± 20	38 ± 19	55 ± 12	53 ± 11
TM8a	H388C (C−)	94 ± 6	94 ± 8	53 ± 6	64 ± 5
TM8a	L390C (C−)	13 ± 3	9 ± 1	24 ± 3	30 ± 3
TM8a	T391C (C−)	25 ± 2	92 ± 8*^b^*	30 ± 2	96 ± 4*^b^*
TM8a	A392C (C−)	11 ± 1	64 ± 5*^b^*	34 ± 4	35 ± 1
TM8a	S393C (C−)	21 ± 3	101 ± 9*^b^*	19 ± 1	63 ± 4*^b^*
TM8a	V394C (C−)	91 ± 10	81 ± 7	28 ± 3	102 ± 4*^b^*
TM8a	M395C (C−)	90 ± 11	107 ± 8	31 ± 2	113 ± 7*^b^*
TM9	I450C (C−)	116 ± 15	126 ± 12	38 ± 2	39 ± 9
TM9	L461C (C−)	87 ± 8	100 ± 10	22 ± 2	22 ± 3
Control	hCNT3	101 ± 6	101 ± 9	20 ± 3	104 ± 9*^b^*
Control	hCNT3C-	109 ± 15	109 ± 16	111 ± 7	101 ± 4

*^a^* Mediated uridine influx in the absence of inhibitor is given in pmol/oocytes·min^−1^ in supplemental Table S1 for each of the individual mutants.

*^b^* Substrate protection.

In HP1, three adjacent residues, Gly^340^, Gln^341^, and Thr^342^, were PCMBS-inhibitable in both Na^+^-containing, H^+^-reduced and Na^+^-free, acidified medium upon conversion to cysteine. Close to Gly^340^, mutant I337C(C−) was PCMBS-inhibitable only in Na^+^-free, acidified medium. In TM7, nine adjacent mutants (I371C(C−), A372C(C−), G373C(C−), S374C(C−), V375C(C−), L376C(C−), G377C(C−), A378C(C−), and I380C(C−)) were PCMBS-inhibitable in both cation conditions, whereas the immediately adjacent residue, Thr^370^, was PCMBS-inhibitable upon conversion to cysteine only in Na^+^-free, acidified medium. One residue that localizes to the predicted extracellular loop following TM7 (Val^384^) was sensitive to PCMBS inhibition upon conversion to cysteine in both Na^+^-containing, H^+^-reduced and Na^+^-free, acidified medium.

PCMBS inhibition was also observed in both Na^+^-containing, H^+^-reduced and Na^+^-reduced, acidified medium for four adjacent mutants in TM8 (L390C(C−), T391C(C−), A392C(C−), and S393C(C−)). In close proximity to this block of residues, three additional mutants (H388C(C−), V394C(C−), and M395C(C−)) were sensitive to PCMBS inhibition only in Na^+^-reduced, acidified medium. Within TM9, two mutants (I450C(C−) and L461C(C−)) were sensitive to PCMBS inhibition only in Na^+^-reduced, acidified medium. None of the residues in the TM8–9 loop, when converted to cysteine in hCNT3(C−), were inhibitable by PCMBS under either cation condition.

### Uridine protection of PCMBS inhibition

Subsequent experiments investigated the ability of extracellular uridine (20 mm) to protect against inhibition by PCMBS for hCNT3(C−) mutants that were inhibited in either or both cation conditions. Results for each individual mutant are presented in Table 1 as percentages of mediated uridine uptake in the absence of PCMBS, and the uridine-protectable residues are highlighted in the hCNT3 topology schematic of Fig. 5.

In HP1, three residue positions exhibited uridine protection from PCMBS inhibition. Q341C(C−) and T342C(C−), which were PCMBS-inhibitable in both Na^+^-containing, H^+^-reduced and Na^+^-free, acidified media, were fully protected by uridine under both cation conditions. In contrast, G340C(C−), which was also PCMBS-inhibitable under both cation conditions, was protected against PCMBS inhibition in Na^+^-containing, H^+^-reduced medium only.

In TM7, five residue positions were uridine-protected. T370C(C−), which was PCMBS-sensitive only in Na^+^-free, acidified medium, exhibited full uridine protection against that inhibition, whereas A372C(C−), G373C(C−), and S374C(C−), which were PCMBS-sensitive in both Na^+^-containing, H^+^-reduced and Na^+^-free, acidified media, were fully protected under both cation conditions. In contrast, I371C(C−), which was inhibited by PCMBS under both cation conditions, was protected by uridine only in Na^+^-containing, H^+^-reduced medium.

In TM8, five residue positions were protected against PCMBS inhibition by uridine. V394C(C−) and M395C(C−), which were PCMBS-inhibited only in Na^+^-reduced, acidified medium, showed full uridine protection, and T391C(C−) and S393C(C−), which were PCMBS-inhibited in both Na^+^-containing, H^+^-reduced and Na^+^-reduced, acidified media, were also fully uridine-protected under both cation conditions. In contrast, A392C(C−), which was also PCMBS-inhibited under both cation conditions, was protected by uridine only in Na^+^-containing, H^+^-reduced medium. None of the PCMBS-sensitive mutants in TM9 was protected by uridine.

### Cation-mediated PCMBS inhibition of hCNT3(C−)

Of the 24 hCNT3(C−) residue positions in the HP1-TM9 region that were PCMBS-sensitive, 17 were inhibited by PCMBS in both cation conditions, whereas 7 were inhibited by PCMBS only in Na^+^-free, acidified medium (Table 1 and Fig. 5). To determine whether access of PCMBS to these residues required cation-induced conformational changes within the protein, each of the single-cysteine mutants at these positions was rescreened for PCMBS inhibition under the original conditions (Na^+^-containing, H^+^-reduced or Na^+^-free, acidified media) and in either Na^+^-free, H^+^-reduced medium (100 mm ChCl, pH 8.5) or Na^+^-containing, acidified medium (100 mm NaCl, pH 5.5). Fluxes of 10 μm uridine were then determined under Na^+^-free, acidified conditions (Table 2).

**Table 2:** **Effects of cations on PCMBS inhibition of hCNT3(C−) single-cysteine mutants** Influx of 10 μm [^3^H]uridine was measured in Na^+^-free acidified medium (100 mm ChCl, pH 5.5) following 10 min of incubation on ice in the absence or presence of PCMBS in either (i) Na^+^-free, H^+^-reduced medium (100 mm ChCl, pH 8.5); (ii) Na^+^-containing, H^+^-reduced medium (100 mm NaCl, pH 8.5); (iii) Na^+^-free, acidified medium (100 mm ChCl, pH 5.5); or (iv) Na^+^-containing, acidified medium (100 mm NaCl, pH 5.5). The values are corrected for basal non-mediated uptake in control water-injected oocytes and are normalized to the corresponding influx of uridine in the absence of inhibitor. Each value is the mean ± S.E. of 10–12 oocytes.

TM		Uptake (100 mm ChCl, pH 5.5)			
		PCMBS in Na^+^-free, H^+^-reduced medium	PCMBS in Na^+^-containing, H^+^-reduced medium	PCMBS in Na^+^-free, acidified medium	PCMBS in Na^+^-containing, acidified medium
		%			
HP1a	I337C (C−)	94 ± 8	104 ± 11	55 ± 9	35 ± 5
HP1a-HP1b loop	G340C (C−)	29 ± 4	18 ± 4	35 ± 5	38 ± 6
HP1a-HP1b loop	Q341C (C−)	48 ± 7	53 ± 6	55 ± 3	51 ± 3
HP1b	T342C (C−)	56 ± 4	53 ± 4	52 ± 3	61 ± 9
TM7a-TM7b loop	T370C (C−)	97 ± 5	91 ± 11	45 ± 3	32 ± 4
TM7a-TM7b loop	I371C (C−)	26 ± 6	23 ± 3	27 ± 8	18 ± 4
TM7a-TM7b loop	A372C (C−)	25 ± 1	19 ± 3	26 ± 3	22 ± 3
TM7a-TM7b loop	G373C (C−)	27 ± 3	28 ± 2	28 ± 3	22 ± 2
TM7a-TM7b loop	S374C (C−)	51 ± 6	35 ± 4	54 ± 8	47 ± 7
TM7b	V375C (C−)	49 ± 6	44 ± 11	37 ± 10	43 ± 4
TM7b	L376C (C−)	27 ± 3	12 ± 4	13 ± 3	33 ± 4
TM7b	G377C (C−)	21 ± 3	21 ± 3	29 ± 4	43 ± 3
TM7b	A378C (C−)	24 ± 4	33 ± 3	24 ± 2	33 ± 2
TM7b	I380C (C−)	50 ± 8	63 ± 5	64 ± 6	73 ± 6
TM7b-TM8a loop	V384C (C−)	47 ± 6	24 ± 4	36 ± 3	45 ± 8
TM8a	H388C (C−)	85 ± 5	92 ± 7	41 ± 3	36 ± 4
TM8a	L390C (C−)	43 ± 4	29 ± 3	26 ± 5	22 ± 2
TM8a	T391C (C−)	15 ± 2	13 ± 1	14 ± 1	13 ± 2
TM8a	A392C (C−)	31 ± 5	42 ± 6	41 ± 6	49 ± 5
TM8a	S393C (C−)	37 ± 3	35 ± 4	29 ± 3	34 ± 3
TM8a	V394C (C−)	60 ± 9	79 ± 6	37 ± 3	29 ± 3
TM8a	M395C (C−)	97 ± 9	87 ± 5	44 ± 5	36 ± 4
TM9	I450C (C−)	88 ± 11	79 ± 9	31 ± 4	29 ± 3
TM9	L461C (C−)	60 ± 3	69 ± 5	20 ± 2	19 ± 3
Control	hCNT3	102 ± 6	107 ± 23	33 ± 3	54 ± 5

All 17 mutants that were inhibited by PCMBS in Na^+^-containing, H^+^-reduced or Na^+^-free, acidified medium were also inhibited by PCMBS in Na^+^-free, H^+^-reduced or Na^+^-containing, acidified medium (Table 2), indicating that PCMBS inhibition was not cation-induced. However, the seven mutants that exhibited PCMBS inhibition only under Na^+^-free, acidified conditions (I337C(C−) in HP1; T370C(C−) in TM7; H388C(C−), V394C(C−), and M395C(C−) in TM8; and I450C(C−) and L461C(C−) in TM9) were unaffected by PCMBS under cation-reduced conditions (Na^+^-free, H^+^-reduced medium) but strongly inhibited by PCMBS in Na^+^-containing, acidified medium as was previously seen for residues Ile^554^, Tyr^558^, and Cys^561^ in TM10 of hCNT3 (28). Thus, access of PCMBS to these residue positions reports a H^+^-induced conformational change of hCNT3.

### SCAM analysis of hCNT1 HP1 and TM7

Complementary to the above studies of hCNT3(C−), we also undertook parallel SCAM analyses of HP1 and TM7 of hCNT1 and of the two equivalent transmembrane regions (HP1 and TM4) of *E. coli* NupC. Wild-type hCNT1 contains 20 endogenous cysteine residues but is insensitive to inhibition by PCMBS under the various cation conditions used in the studies with hCNT3(C−) (17, 28). This enabled use of the wild-type protein as a template for the construction of single-cysteine mutants to minimize the possibility that results obtained for the same TMs in hCNT3(C−) were influenced by engineering the removal of its endogenous cysteine residues. It was additionally hoped that a comparison of results for Na^+^-specific hCNT1 with those for Na^+^- and H^+^-dependent hCNT3 would identify features associated with the different cation-coupling characteristics of these transporters.

**Figure 6.**
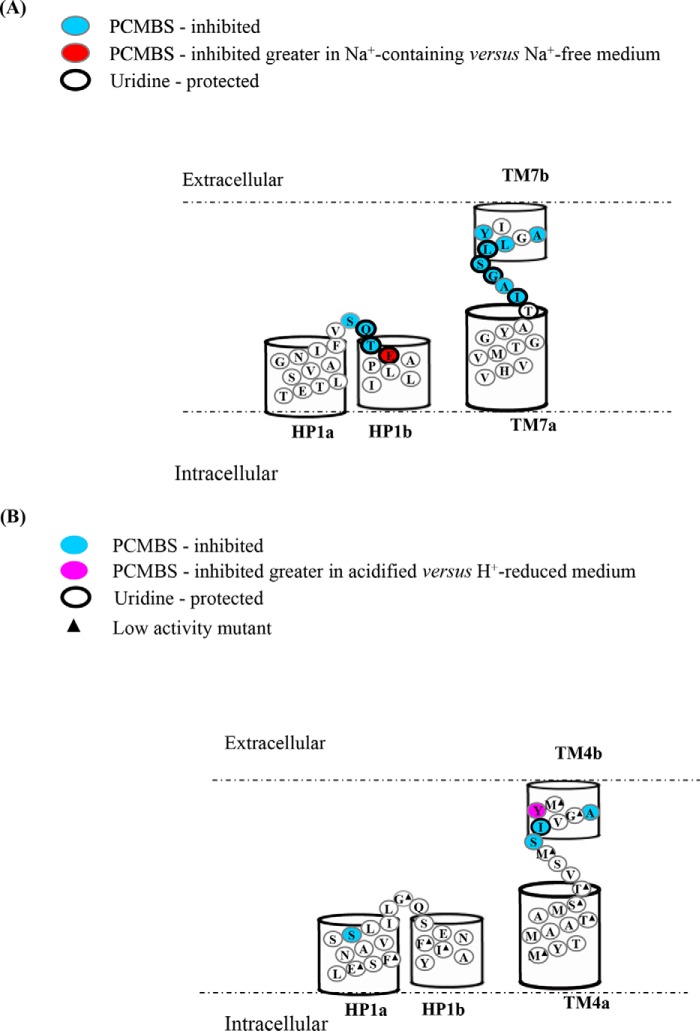
*A*, hCNT1 HP1a, HP1b, TM7a, and TM7b depicting PCMBS-inhibited and uridine-protected residues. hCNT1 TMs HP1a, HP1b, TM7a, and TM7b PCMBS-sensitive residues are highlighted in *blue*, and the single residue exhibiting greater inhibition in Na^+^-containing *versus* Na^+^-free medium is indicated in *red*. Residues protected from PCMBS inhibition by excess unlabeled uridine are *outlined* in *black*. Corresponding numerical values are given in supplemental Tables S2 and S3. *B*, NupC(C−) HP1a, HP1b, TM4a, and TM4b region depicting PCMBS-inhibited and uridine-protected residues. NupC(C−) mutants exhibiting inhibition of uridine uptake following incubation with PCMBS in both acidified and H^+^-reduced media are indicated in *blue*. The single residue exhibiting greater inhibition in acidified *versus* H^+^-reduced medium is indicated in *pink*. The residue protected from PCMBS inhibition by excess unlabeled uridine is *outlined* in *black*. Low-activity mutants are indicated by *black triangles.* Corresponding numerical values are given in supplemental Tables S5 and S6.

**Figure 7.**
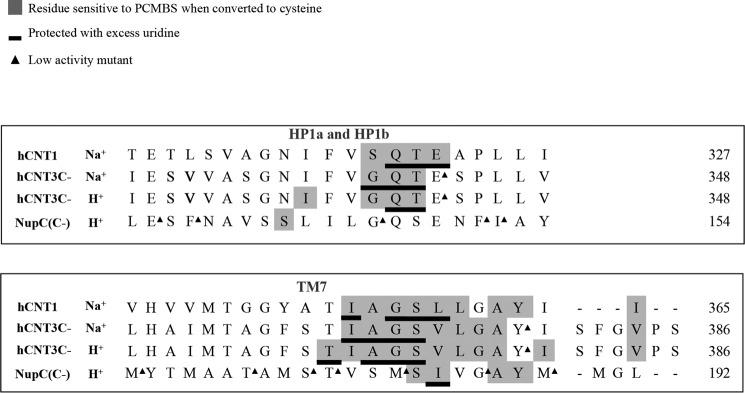
**Comparison of PCMBS inhibition and substrate protection of residues in the HP1a, HP1b, TM4a, and TM4b region of NupC with the corresponding HP1a, HP1b, TM7a, and TM7b regions of hCNT3(C−) and hCNT1.** Residues sensitive to PCMBS inhibition are highlighted in *gray*, and those protected with excess 20 mm uridine are *underlined*. Low-activity mutants are indicated by *black triangles*. Some constructs were unavailable for testing and are denoted with a *hyphen*.

The 43 residues of hCNT1 spanning the region between and including HP1 and TM7 that were investigated in the present study are highlighted in supplemental Fig. S1. Single-cysteine mutants were scanned for functional activity in Na^+^-containing, H^+^-reduced medium (supplemental Table S2) and tested for PCMBS inhibition and uridine protection under the same conditions (supplemental Table S3). To determine whether access of PCMBS required Na^+^-induced conformational changes, inhibitor-sensitive mutants were rescreened for PCMBS inhibition under either Na^+^-containing, H^+^-reduced or Na^+^-free, acidified conditions (supplemental Table S4). Media were buffered at pH 8.5 to enable direct comparison with data obtained for hCNT3(C−). Control experiments at pH 7.5 yielded essentially identical results (data not shown). Results from the experiments are summarized in the topology schematic of Fig. 6*A*, and compared with hCNT3(C) in the sequence alignment of Fig. 7. Because none of the 43 hCNT1 mutants examined exhibited uridine uptake values of <0.1 pmol/oocyte·min^−1^, it was possible to test all 43 individual cysteine mutants for sensitivity to PCMBS inhibition.

In HP1 of hCNT1, PCMBS inhibition was evident for residue positions Ser^319^, Gln^320^, Thr^321^, and Glu^322^. Of these, Gln^320^, Thr^321^, and Glu^322^ showed uridine protection (Fig. 6*A*). This clustering of adjacent PCMBS-inhibitable residues was similar to hCNT3(C−) (Figs. 5 and 7) and inconsistent with the patterning anticipated for a conventional α-helix.

In TM7 of hCNT1, six adjacent residues were sensitive to PCMBS inhibition when mutated to cysteine (Ile^350^, Ala^351^, Gly^352^, Ser^353^, Leu^354^, and Leu^355^). Of these, mutants I350C, G352C, S353C, and L354C showed uridine protection. Again, the clustering of adjacent PCMBS-sensitive residues was similar to hCNT3(C−) and inconsistent with a conventional α-helix. Similarities between the two transporters also extended to the presence of a single PCMBS-sensitive residue in the putative external loop exiting TM7 (Ile^363^ in hCNT1 and Val^384^ in hCNT3(C−)) (Table 1 and supplemental Table S3). Residues that were sensitive to PCMBS inhibition in the presence of Na^+^ were also inhibited by PCMBS in its absence (supplemental Table S4), indicating that access of PCMBS to hCNT1 inhibitor-sensitive residues did not require Na^+^.

### SCAM analysis of NupC HP1 and TM4

Extending the analysis to H^+^-specific NupC explored whether features revealed in hCNT1/3 were universal to all CNT family members or possibly related to the Na^+^ coupling characteristics of the human proteins. In initial experiments, the single endogenous cysteine residue present in wild-type NupC (Cys^96^) was replaced with either serine or alanine to produce cysteine-less NupC constructs C96S and C96A, respectively. Produced in *Xenopus* oocytes, C96S exhibited transport characteristics similar to those of wild-type NupC, but with lower functional activity, exhibiting a moderate ∼3-fold increase in uridine apparent *K_m_*, a kinetic deficit that was overcome by conversion of Cys^96^ instead to alanine (data not shown). NupC C96A (hereafter referred to as NupC(C−)), therefore, was used as template for the construction of single-cysteine mutants.

The 45 NupC(C−) residues spanning the region between and including HP1 and TM4 of NupC (corresponding to hCNT1/3 HP1 and TM7) that were investigated are highlighted in supplemental Fig. S2. Single-cysteine mutants were scanned for functional activity in Na^+^-free, acidified medium (supplemental Table S5) and tested for PCMBS inhibition and uridine protection under the same conditions (supplemental Table S6). To determine whether access of PCMBS required H^+^-induced conformational changes, inhibitor-sensitive mutants were rescreened for PCMBS inhibition under either Na^+^-free, acidified or Na^+^-free, H^+^-reduced conditions (supplemental Table S7). Results from the experiments are summarized in the topology schematic of Fig. 6*B* and compared with hCNT3(C−) and hCNT1 in the sequence alignment of Fig. 7.

Of the 43 NupC(C−) mutants examined, 12 exhibited uridine uptake values of <0.05 pmol/oocyte·10 min^−1^ and were excluded from further analysis (supplemental Table S5). Five of these were in HP1 (E135C(C−), F137C(C−), G146C(C−), F151C(C−), and I152C(C−)), and 7 were in TM4 (M166C(C−), T172C(C−); S175C(C−), T176C(C−), M179C(C−), G183C(C−), and M186C(C)). Mutagenesis of NupC(C−) therefore resulted in a higher percentage of non-functional mutants when compared with mutagenesis of hCNT3(C−) or hCNT1. This may, in part, reflect production in a eukaryote heterologous expression system and the attendant lower overall transport activity exhibited by the bacterial transporter. However, as shown in the sequence alignment of Fig. 7, most of the non-functional NupC(C−) mutants clustered in the same TM regions shown to be functionally important for hCNT3(C−) and hCNT1. For hCNT3(C−), H^+^ coupling is generally more sensitive to mutation than Na^+^-coupling, and because NupC is exclusively H^+^-dependent, this might also be a contributing factor to the larger number of non-functional NupC mutants.

In HP1 of NupC(C−), PCMBS inhibition was evident only for residue position Ser^142^, and this inhibition was not uridine-protectable. This residue is not part of the cluster of adjacent residues found to be PCMBS-sensitive in hCNT3(C−) and hCNT1. It is, however, centrally positioned in the TM and precedes by one position a residue in hCNT3(C−) (Ile^337^) that was sensitive to PCMBS inhibition under Na^+^-reduced, acidified conditions.

In TM4 of NupC(C−), two pairs of adjacent mutants, S180C(C−)/I181C(C−) and A184C(C−)/Y185C(C−), were inhibited by PCMBS. Only I181C(C−) was protected by uridine. The corresponding residues in hCNT3(C) and hCNT1 are part of the cluster of adjacent residues that were also sensitive to PCMBS inhibition.

Residues that were sensitive to PCMBS inhibition in the presence of H^+^ were also inhibited by PCMBS in its absence (supplemental Table S7). Access of PCMBS to NupC inhibitor-sensitive residues did not therefore require H^+^. One mutant, Y185C(C−), was more sensitive to PCMBS inhibition under acidified conditions but moderately inhibited by PCMBS in H^+^-reduced medium (supplemental Table S7).

### Permeant selectivity of hCNT3(C−) HP1 and TM7 mutants

**Figure 8.**
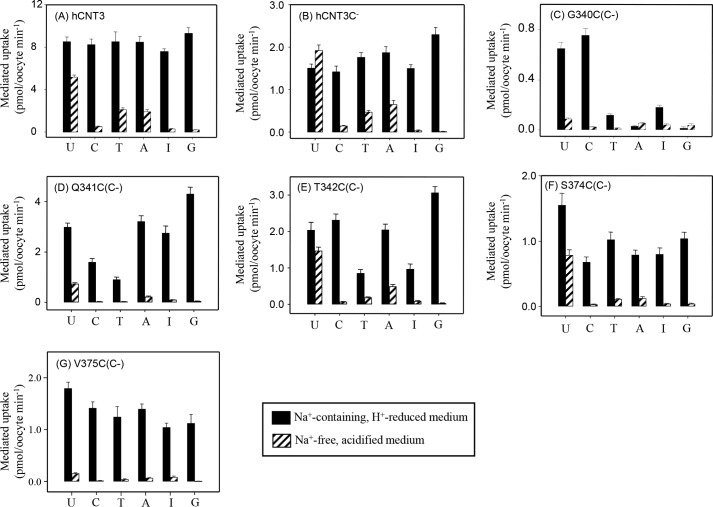
**Permeant selectivity of wild-type hCNT3, hCNT3(C−) and hCNT3(C−) single-cysteine mutants G340C(C−), Q341C(C−), T342C(C−), S374C(C−), and V375C(C−).** Oocytes producing wild-type hCNT3 (*A*), hCNT3(C−) (*B*), or hCNT3(C−) single-cysteine mutants (*C–G*) were incubated with 10 μm nucleosides: *U*, uridine; *C*, cytidine; *T*, thymidine; *A*, adenosine; *I*, inosine; *G*, guanosine. The initial rates of uptake were measured in either Na^+^-containing, H^+^-reduced or Na^+^-free, acidified media, at 20 °C. The data are presented as mediated transport, calculated as uptake in RNA-injected oocytes minus uptake in water-injected oocytes. Each value is the mean ± S.E. of 10–12 oocytes.

HP1 and TM7 of hCNT1/2 contain residues that determine the nucleoside preferences of the two transporters (35, 36). A final series of experiments investigated residues within these regions of hCNT3(C−) for potential roles in nucleoside selectivity (Fig. 8). The uridine-protected subset of PCMBS-sensitive hCNT3(C−) mutants were subjected to transport experiments involving a panel of radiolabeled purine and pyrimidine nucleosides (each at 10 μm). Of the eight hCNT3(C−) cysteine mutants tested, five showed significantly different nucleoside uptake profiles compared with those of wild-type hCNT3 and hCNT3(C−).

As previously reported, wild-type hCNT3 and hCNT3(C−) exhibited broad transport preference for both purine and pyrimidine nucleosides in Na^+^-containing, H^+^-reduced medium (20, 27) (Fig. 8, *A* and *B*). Both also exhibited the characteristic narrowing of nucleoside preference under Na^+^-free, acidified conditions (uridine > thymidine, adenosine > cytidine, and inosine > guanosine (20, 27)).

Three of the mutants with altered permeant selectivities were located within HP1 (G340C(C−), Q341C(C−), and T342C(C−)), and two were located in TM7 (S374C(C−) and V375C(C−)). Of these, G340C(C−) and V375C(C−) had the greatest differences compared with hCNT3 and hCNT3(C−). Relative to uridine, G340C(C−) exhibited reduced uptake of thymidine, adenosine, inosine, and guanosine in Na^+^-containing, H^+^-reduced medium, whereas uptake was greatly reduced, although measurable, for all nucleosides tested in Na^+^-free, acidified medium (Fig. 8*C*). Similar to hCNT3 and hCNT3(C−), V375C(C−) exhibited broadly selective uptake of both purine and pyrimidine nucleosides in Na^+^-containing, H^+^-reduced medium, but only very low uptake of uridine, thymidine, adenosine and inosine, and no detectable transport of cytidine and guanosine in Na^+^-free, acidified medium (Fig. 8*G*).

Immediately adjacent to residue Gly^340^ in HP1, mutants Q341C(C−) and T342C(C−) showed (i) reduced uptake of cytidine and thymidine (Q341C(C−)) or thymidine and inosine (T342C(C−)) in Na^+^-containing, H^+^-reduced medium and (ii) reduced uptake of uridine, cytidine, thymidine, and adenosine (Q341C(C−)) or cytidine and thymidine (T342C(C−)) in Na^+^-free, acidified medium (Fig. 8, *D* and *E*).

Adjacent to Val^375^ in TM7, mutant S374C(C−) showed a normal uptake profile in Na^+^-containing, H^+^-reduced medium but reduced uptake of uridine, cytidine, thymidine, and adenosine in Na^+^-free, acidified medium (Fig. 8*F*). Gly^340^, Gln^341^, Ser^374^, and Val^375^ in hCNT3 correspond to the two adjacent pairs of HP1 and TM7 residues in hCNT1 that have previously been shown to determine the differences in permeant selectivity between that transporter and hCNT2 (35, 36).

## Discussion

The present study investigated the IH2-TM9 region of hCNT3(C−) and, for comparison, HP1 and TM7 of hCNT1 and HP1 and TM4 of *E. coli* NupC(C−). Together with previous studies of IH3-TM11 of hCNT3(C−) (28, 29), the present investigation completed SCAM analysis of the entire transport domain of hCNT3 by systematic analysis of functional and structural relationships among 305 consecutive residues of hCNT3 encompassing residues Met^308^ to Thr^615^. Consistent with the structural model of vcCNT topology (21), all of the PCMBS-inhibitable residues in TMs IH2-TM9 lie exofacially within the membrane in positions likely to be accessible to the extracellular medium and available for PCMBS binding (Fig. 5).

The patterns of PCMBS inhibition reported here for IH2-TM9 provide important functional evidence of extended structures resembling the discontinuous membrane helices evident not only in vcCNT, but also in the crystal structures of other Na^+^-coupled bacterial transporters, including *Aquifex aeolicus* LeuT_Aa_ (37), *Vibrio parahemeolyticus* SGLT (38), *E. coli* NhaA (39), *Microbacterium liquefaciens* NCS1 (40), and *Pyrococcus horikoshii* GltPh (41). Reviewed by Screpanti and Hunte (42) and Krishnamurthy *et al.* (43), such discontinuous membrane helices play important mechanistic roles in ion and permeant recognition, binding, and translocation in secondary active transporters.

Introduction of cysteine residues into HP1, TM7, and TM8 of hCNT3(C−) revealed multiple examples of PCMBS-sensitive, uridine-protected residue positions with altered Na^+^:H^+^ uptake ratios and, in several cases, altered permeant selectivities, indicating that these regions form part of a common nucleoside/cation translocation pore and that residues contributed by TMs 7 and 8 have locations within or closely adjacent to neighboring and functionally integrated nucleoside and cation-binding pockets. In TM9, in contrast and in keeping with its structural role as a linker between the two transport subdomains of hCNT3, only two residues were moderately inhibited by PCMBS, and neither was protected by uridine. Residue positions that, by homology with vcCNT, potentially have specific roles in cation and nucleoside binding are discussed separately under “Mechanistic implications.”

The present results also contribute insight into cation-dependent conformations adopted by the exofacially facing form of the protein. In contrast to Na^+^-specific hCNT1 and hCNT2, hCNT3 mediates both Na^+^- and H^+^-coupled nucleoside cotransport (11, 15, 17–20). The cation:nucleoside stoichiometry for hCNT3 H^+^-coupled transport is 1:1 compared with 2:1 for Na^+^, and when both cations are present, charge/uptake experiments suggest that hCNT3 binds one Na^+^ and one H^+^ (19, 20). The nucleoside and nucleoside drug selectivity patterns of hCNT3 in the presence of H^+^ also differ from those in the presence of Na^+^ (11, 20). Previously, mutation of hCNT3 Cys^561^ in TM10 was reported to alter Na^+^ and H^+^ kinetics and, together with Tyr^558^ and Ile^554^, to form a face of the helix that becomes extracellularly accessible to PCMBS only in the presence of H^+^, thus reporting a H^+^-dependent conformation of the protein (27). Building upon these observations, the present results identified seven additional residues in IH2-TM9 whose accessibilities to PCMBS similarly report a H^+^-dependent conformation of the protein (Table 2 and Fig. 5) and some of which (*e.g.* H^+^-specific TM12 Ile^554^/Tyr^558^/Cys^561^) are clustered together and may involve subdomains within TMs. Most notable in this regard are three residues in TM8 (His^388^, Val^394^, and Met^395^). Other potential conformational differences are even more subtle. The *arrows* in Fig. 5, for example, identify residues in HP1, TM7, and TM8 that were PCMBS-sensitive in both Na^+^-containing, H^+^-reduced and Na^+^-free, acidified media but were uridine-protected only in Na^+^-containing, H^+^-reduced medium.

### hCNT1 and NupC(C−)

Investigation of HP1 and TM7 of wild-type hCNT1 confirmed the SCAM analysis of the corresponding region of hCNT3(C−) and, in so doing, eliminated any possibility that results for hCNT3(C−) were influenced by removal of endogenous cysteine residues from the transporter. Corresponding SCAM analysis of HP1 and TM4 of H^+^-specific *E. coli* NupC(C−) revealed both similarities and differences between the bacterial transporter and Na^+^-specific hCNT1 and Na^+^- and H^+^-dependent hCNT3.

Although the PCMBS-sensitive residues in NupC(C−) fell within the regions of proposed helical discontinuity in hCNT3(C−) and hCNT1, there were not as many, possibly because of the larger number of functionally inactive mutants that may have an origin in the potential hydrophobic mismatch resulting from insertion of a bacterial protein into a eukaryote membrane environment. Nevertheless, the results provide reinforcing evidence that NupC(C−) HP1 and TM4 contribute to the CNT translocation pore. Within TM4, PCMBS sensitivity of one of the residues (Tyr^185^) reported a H^+^-dependent conformation of the protein.

### Mechanistic implications

**Figure 9.**
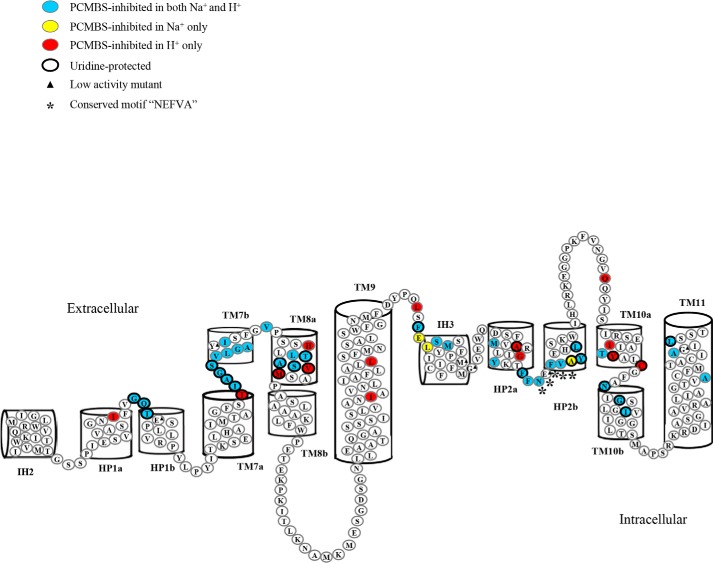
**Proposed C-terminal topology of hCNT3.** hCNT3(C−) mutants exhibiting inhibition of uridine uptake following incubation with PCMBS in both Na^+^-containing, H^+^-reduced and Na^+^-free, acidified media are indicated in *blue*, those that were inhibited only in Na^+^-containing, H^+^-reduced medium are indicated in *yellow*, and those that were inhibited only in Na^+^-free, acidified medium are indicated in *red*. Residues protected from PCMBS inhibition by excess unlabeled uridine are *outlined* in *black*. The *asterisks* represents residues that form the conserved CNT family (G/A)*X*K*X*_3_NEFVA(Y/M/F) motif. Low-activity mutants are indicated by a *solid triangle*. Residues of hCNT3(C−) mutants in IH3, HP2a, HP2b, TM10a, TM10b, and TM11 exhibiting inhibition of uridine uptake following incubation with PCMBS and residues protected from PCMBS inhibition by excess unlabeled uridine have been previously published (Fig. 5 in Ref. 28).

In addition to the new data for hCNT3 IH2-TM9, Fig. 9 also incorporates our previous SCAM analysis of hCNT3 TMs IH3-TM11. The functional identification of extended regions of polypeptide in hCNT3(C−) HP2 (28) and, now, HP1, TM7, and TM8 strongly supports the central role of these TMs in formation of the CNT nucleoside/cation-binding pocket(s) and common translocation pore that also has contributions from HP2, TM10, and TM11 (26–29, 33, 35, 36).

Table 3 lists the major specific vcCNT residues implicated in Na^+^ and uridine binding and, for comparison, the homologous residues in hCNT3 (and hCNT1 and NupC). The residues mutated in the present study, or previously, are shown in bold type. Cysteine mutants of each of the four hCNT3 residues potentially involved in Na^+^ binding were functional and, in three of the four cases, exhibited altered Na^+^:H^+^ uridine uptake ratios, being either Na^+^-preferring (Asn^336^ and Val^339^) or H^+^-preferring (Thr^370^). Two were PCMBS-sensitive (Thr^370^ and Ile^371^), one in the presence of H^+^ only (Thr^370^). Both of these were uridine-protected.

**Table 3:** **vcCNT residues and corresponding hCNT3, hCNT1, and NUPC residues implicated in Na^+^ and uridine binding (21, 55)** Residues mutated in the present study and in Refs. 28, 29, 32, and 33 are in bold type.

Type of interaction	vcCNT	hCNT3	hCNT1	NupC
Uridine-binding site				
Side chain	Gln^154^	**Gln^341^**	**Gln^320^**	**Gln^147^**
Side chain	Thr^155^	**Thr^342^**	**Thr^321^**	**Ser^148^**
Side chain	Glu^156^	**Glu^343^*^a^***	**Glu^322^**	**Glu^149^**
Side chain	Glu^332^	**Glu^519^*^a^***	**Glu^498^**	Glu^321^
Side chain	Asn^368^	**Asn^565^**	Asn^544^	Asn^352^
Side chain	Ser^371^	**Ser^568^*^a^***	Ser^547^	Ser^355^
π-π and CH-π	Phe^366^	**Phe^563^*^a^***	Phe^542^	Phe^350^

Sodium-binding site				
Backbone carbonyl group	Asn^149^	**Asn^336^*^b^***	**Asn^315^**	**Ser^142^**
Side chain hydroxyl group	Asn^149^	**Asn^336^*^b^***	**Asn^315^**	**Ser^142^**
Backbone carbonyl group	Val^152^	**Val^339^**	**Val^318^**	**Leu^145^**
Side chain hydroxyl group	Ser^183^	**Thr^370^**	**Thr^349^**	**Thr^176^*^a^***
Backbone carbonyl group	Ile^184^	**Ile^371^**	**Ile^350^**	**Val^177^**

*^a^* Non-functional mutants.

*^b^* Proton-dependent flux abolished.

In hCNT1, all four of the corresponding residue mutations were functional. One had low activity (Thr^349^), and one (Ile^350^) was inhibited by PCMBS and uridine-protected. In NupC, three of the four corresponding residue mutations were also functional (Ser^142^, Leu^145^, and Val^177^), with one having low but measurable activity (Val^177^). The non-functional residue position was Thr^176^. Only the Ser^142^ residue position was inhibited by PCMBS, and this inhibition was not protected by uridine.

Of the seven main hCNT3 residues potentially involved in uridine binding, four were investigated in the present study (Gln^341^, Thr^342^, Glu^343^, and Phe^563^), and three were investigated previously (Glu^519^, Asn^565^ and Ser^568^) (28). As a group, four were functionally inactive when converted to cysteine (Glu^343^, Glu^519^, Ser^568^, and Phe^563^), the two glutamate substitutions being present at cell surfaces in reduced amounts. Of the three residue positions that retained function following conversion to cysteine (Gln^341^, Thr^342^, and Asn^565^), two exhibited altered (Na^+^-preferring) Na^+^:H^+^ uridine uptake ratios (Gln^341^ and Asn^565^). All were PCMBS-sensitive under both cation conditions and uridine-protected. Mutation of Gln^341^ and Thr^342^ both resulted in changes in permeant selectivity, Gln^341^ corresponding to hCNT1 Gln^320^, a residue previously implicated in hCNT1/2 permeant selectivity (36).

In hCNT1, mutation of the three residues corresponding to hCNT3 Gln^341^, Thr^342^, and Glu^343^ yielded proteins with either normal (Gln^320^) or low but measurable transport activity (Thr^321^ and Glu^322^). Similar to hCNT3, all were PCMBS-sensitive and uridine-protected. Likewise, for Na^+^ binding, all four of the corresponding hCNT1 residue mutations were again functional. One had low activity (Thr^349^), and one (Ile^350^) was inhibited by PCMBS and uridine-protected.

In NupC, mutation of the three residues corresponding to hCNT3 Gln^341^, Thr^342^, and Glu^343^ similarly yielded proteins with either normal (Ser^148^) or low but measurable transport activity (Gln^147^ and Glu^149^). Different from hCNT1/3, however, none were inhibited by PCMBS. Generally, therefore, the present and previous SCAM and other mutagenesis results are consistent with the Table 3 listing of residues potentially implicated in Na^+^ and uridine binding.

**Figure 10.**
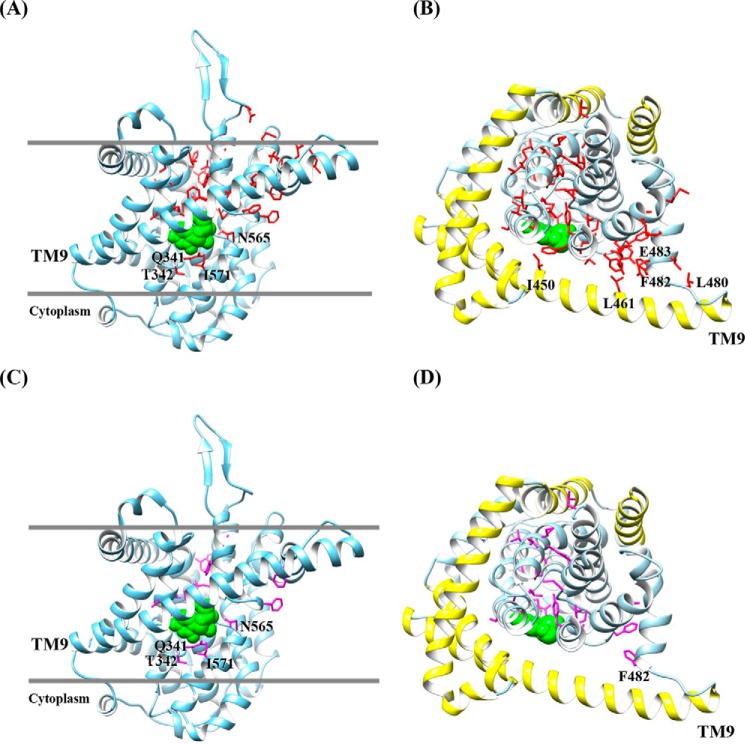
**Homology model of hCNT3.** Shown are cartoon representations of hCNT3 3D models based upon the crystal structure of the bacterial nucleoside transporter vcCNT (Protein Data Bank accession code 3TIJ) using the program SWISS-MODEL (56). Molecular graphics and analyses were performed with the UCSF Chimera package (57). *A* and *C*, models of hCNT3 viewed parallel to the membrane. The extracellular boundaries of the hydrophobic core of the bilayer predicted using the PPM server are shown as *gray lines* (58). *B* and *D*, models of hCNT3 viewed from the extracellular surface of the membrane. The outer scaffold domain of hCNT3 (TM4, TM5, IH1, TM6, and TM9) is shown in *yellow*. For clarity the loop linking HP2 and TM10 is not shown. Side chains of PCMBS-sensitive residues (*A* and *B*) are shown in *red*. Side chains of PCMBS-sensitive and uridine-protected residues (*C* and *D*) are shown in *purple*. The bound uridine molecule is shown in space filling representation (*green*).

Extending the linear topology model of hCNT3 shown in Fig. 9, we also constructed a 3D structural homology model of hCNT3 based upon the inward-facing occluded vcCNT crystal structure (Fig. 10). In Fig. 10, *A* and *C* show hCNT3 viewed parallel to the membrane, whereas *B* and *D* view hCNT3 from the extracellular surface. Highlighted in *yellow* in Fig. 10 (*B* and *D*) is the scaffold domain of hCNT3 (TM4, TM5, IH1, TM6, and TM9). Highlighted in *blue* in Fig. 10 (*B* and *D*) is the corresponding transport domain of hCNT3 comprising two subdomains (IH2, HP1, TM7, and TM8) and (IH3, HP2, TM10, and TM11). PCMBS is membrane-impermeable and targets extracellularly accessible residues. As such, side chains of amino acids in *red* reflect PCMBS-sensitive residues accessible to inhibition in the outward-facing conformation of the transporter (Fig. 10, *A* and *B*), whereas those in *purple* show the subset of these residues likely to be within or closely adjacent to the outward-facing permeant binding pocket (Fig. 10, *C* and *D*). Fig. 10*C* shows four PCMBS-sensitive and uridine-protected amino acid residues (Gln^341^, Thr^342^, Asn^565^, and Ile^571^) located deeper and closer to the cytoplasmic side of the membrane but still exposed to the extracellular medium during the transport cycle. Gln^341^ and Thr^342^ are located in the flexible, unwound region of the HP1, whereas Asn^565^ and Ile^571^ are in the hinge region of TM10, both regions being part of the transport domain for permeant binding.

Of the 305 residues studied, a total of 53 were PCMBS-sensitive. Of these, 25 were uridine-protected. None of the PCMBS-sensitive residues were located in loops (except that linking TM9 and IH3), and the majority (87%) were located in the transport domain. Overall, the clustering of PCMBS-sensitive residues around the bound uridine molecule suggests that the permeant-binding pockets in the outward- and inward-facing conformations of the transporter are more similar than different. This and the interrelationship between the surrounding outer scaffold and inner transport domains as viewed from the extracellular membrane surface are reminiscent of an emerging novel elevator-type mechanism of transporter function very different from conventional alternating access rocking models of membrane transport. Whereas the latter feature rocker bundle or rocker switch mechanisms to alternately expose bound permeant to extracellular and intracellular membrane surfaces, the elevator mechanism involves a static scaffold domain and a mobile transport domain (44). To provide alternating access, a relatively unchanged transport domain undergoes large downward or upward motions along the scaffold domain. First proposed for GltPh, a number of other Na^+^-coupled transporters are now thought to function in a similar manner (45–49). From its original inward-facing conformation, vcCNT has also been proposed to utilize an elevator-type mechanism for permeant translocation (21). Consistent with this, scaffold domain TM9 has two PCMBS-sensitive residues (Ile^450^ and Leu^461^), neither of which are uridine-protected, but both of which have locations potentially able to interfere with elevator movement of the transport domain. Similarly, three PCMBS residues (Leu^480^, Phe^482^, and Glu^483^) were identified in the hinge region linking TM9 of the scaffold domain to IH3 of the transport domain.

A recently developed repeat-swap outward-facing homology model of vcCNT (protein model database (PMDB) identifier PM0080188) is also consistent with an elevator-type transport mechanism (50). Derived from this structure, we have generated a corresponding outward-facing model of hCNT3 (supplemental Fig. S4). Relative to its inward-facing conformation, the repeat-swap outward-facing model of hCNT3 shows the bound permeant uridine to be elevated and more accessible to the extracellular milieu.

### Conclusions

The present analysis of the IH2 to TM9 region of hCNT3(C−) completes a systematic SCAM analysis of the entire transport domain of the protein. The present study also describes the successful use of the *Xenopus* oocyte heterologous expression system to undertake PCMBS SCAM analysis of wild-type hCNT1 and *E. coli* NupC(C−). The results highlight functionally important residues and support the structural topology model for hCNTs predicted from the crystal structure of vcCNT.

The vcCNT structure is in an inward-facing occluded form, with access to the uridine-binding site completely blocked from the periplasmic surface of the membrane and hindered from the cytoplasmic surface. Transport must therefore involve transitions between this state and conformations in which the binding site is fully exposed either to the periplasmic space or cytoplasm. The specific nature of such transitions remains unclear, although as shown here and previously (28, 29), a number of sites in hCNT3 have been shown to exhibit cation-induced changes in protein conformation. Also, residues sensitive to inhibition by PCMBS must be accessible to the external medium, with those exhibiting uridine protection potentially located within or closely adjacent to the outward-facing conformation of the nucleoside-binding pocket.

hCNT3 has two cation-binding sites, one of which is Na^+^-specific and one of which also accepts H^+^. Given that the vcCNT crystal structure has a single bound Na^+^, it remains to be determined with which of the two hCNT3 cation-binding sites it is equivalent. Because mutation of hCNT3 residues predicted to be part of that Na^+^-binding site also showed changes in H^+^-dependent uridine transport, these residues possibly contribute to the Na^+^/H^+^-binding site of hCNT3. On the other hand, other regions of hCNT3 also exhibit residues whose mutation influence both Na^+^ and H^+^ coupling (51). Finally, the data are consistent with an elevator-type mechanism of membrane transport, in which permeant (and cation) translocation are achieved through a static scaffold domain and a mobile transport domain.

## Experimental procedures

### Constructs

Molecular cloning of hCNT1 (15), hCNT3 (11), and NupC (25) (GenBank^TM^ accession numbers U62968, AF305210, and NC000913, respectively) has been previously described. The hCNT3 cDNA provided the template for the construction of a cysteine-less version of hCNT3 (hCNT3(C−)) in which all 14 endogenous cysteine residues were converted to serine (26). Similarly, the NupC cDNA provided the template for construction of a cysteine-less version of NupC (NupC(C−) or C96A) in which the single endogenous cysteine residue in the protein (Cys^96^) was converted to alanine. hCNT3(C−), NupC(C−), and wild-type hCNT1 were transferred into the *Xenopus* oocyte expression vector pGEM-HE. By providing additional 5′- and 3′-untranslated regions from the *Xenopus* β-globin gene flanking the multiple cloning site, pGEM-HE gives enhanced production and functional activity of heterologous proteins expressed in *Xenopus* oocytes (52).

### Site-directed mutagenesis and production in Xenopus laevis oocytes

In hCNT3(C−), NupC(C−), or wild-type hCNT1, residues were individually converted into cysteine using the QuikChange® site-directed mutagenesis kit (Stratagene). Constructs were analyzed in both directions by *Taq* dyedeoxy-terminator cycle sequencing to confirm that the anticipated mutation had been correctly introduced. Plasmid DNA was linearized with Nhe1 and transcribed with T7 polymerase using the mMESSAGE mMACHINE^TM^ (Ambion) transcription system. Defolliculated stage VI *Xenopus* oocytes were microinjected with 20 nl of water or 20 nl of water containing capped RNA transcripts (20 ng) and incubated in modified Barth's medium (changed daily) at 18 °C for 72 h prior to assay of transport activity.

### Flux assays and transport inhibition

Transport assays were performed as described previously (13, 29, 53). Groups of 12 oocytes were incubated at room temperature (20 °C) in 200 μl of transport medium containing either 100 mm NaCl or ChCl, and 2 mm KCl, 1 mm CaCl_2_, 1 mm MgCl_2_, and 10 mm HEPES (pH 8.5) or MES (pH 5.5). Uptake was traced with ^14^C- or ^3^H-labeled nucleosides (1 or 2–4 μCi/ml, respectively) (Moravek Biochemicals, Sigma, or GE Healthcare) at a concentration of 10 μm for hCNTs or 1 μm for NupC. Transport medium for adenosine uptake experiments also contained 1 μm deoxycoformycin to inhibit adenosine deaminase activity. All uptake values represent initial rates of transport (influx) determined using an incubation period of 1 min for hCNTs and 10 min for NupC (11, 15, 17, 25). At the end of the incubation period, extracellular label was removed by seven rapid washes in ice-cold Na^+^-free (choline chloride) transport medium (100 mm ChCl, pH 7.5), and individual oocytes were dissolved in 1% (w/v) SDS for quantitation of oocyte-associated radioactivity by liquid scintillation counting (LS 6000 IC; Beckman). Also in a volume of 200 μl, oocytes were treated on ice for 10 min with 200 μm PCMBS. Excess PCMBS was removed by three washes with ice-cold transport medium before the assay of transport activity. As shown in supplemental Fig. S3, a 10-min exposure of wild-type hCNT3 was sufficient to cause maximum inhibition of transport and, as a control, mutant hCNT3(C−) T370C exhibited a similar time course of inhibition (data not shown). In protection experiments, unlabeled uridine (20 mm) was included along with PCMBS (28, 29, 34, 35). The flux values shown represent mediated transport corrected for basal uridine uptake measured in control water-injected oocytes and are the means ± S.E. of 10–12 oocytes. Uptake values for hCNT3(C−) and hCNT1 or NupC(C−) are reported in units of pmol/oocyte·min^−1^ or pmol/oocyte·min^−1^, respectively.

### Cell-surface expression

The presence of recombinant hCNT3(C−) and hCNT3(C−) mutant proteins at oocyte cell surfaces was determined by labeling intact oocytes with EZ-Link sulfo-NHS-LC-biotin (Pierce) followed by isolation of the resulting biotinylated plasma membrane proteins using immobilized streptavidin resin (Pierce) according to the manufacturer's instructions. For immunoblotting, solubilized proteins (one oocyte/lane) were resolved on NuPAGE® Novex® Bis-Tris mini gels (Invitrogen). The electrophoresed proteins were transferred to polyvinylidene difluoride membranes (GE Healthcare) and probed with affinity-purified anti-hCNT3_45–69_ polyclonal antibodies (54). Blots were then incubated with horseradish peroxidase-conjugated anti-rabbit antibodies (Pierce) and developed with enhanced chemiluminescence reagents (Pierce).

## Author contributions

R. M. conducted the flux and transport inhibition assays and analyzed the results. R. M. and S. Y. M. Y. undertook the surface expression assays, and A. M. L. N. prepared the mutants. C. E. C. and J. D. Y. conceived the project, interpreted the data, and, with R. M. and S. Y. M. Y., wrote the manuscript.

## Supplementary Material

Supplemental Data

## Footnotes

2 The abbreviations used are:

ENT: equilibrative nucleoside transporter
CNT: concentrative nucleoside transporter
TM: putative transmembrane domain
SCAM: substituted cysteine accessibility method
PCMBS: *p*-chloromercuribenzene sulfonate
vcCNT: CNT from *V. cholerae*
IH: interfacial helix
HP: hairpin.

## Note added in proof

Complementing the inward-facing conformation of vcCNT, Lee and co-workers now report the crystal structures of the inward-facing, intermediate and outward-facing states of a bacterial CNT from *Neisseria wadsworthii* (CNT_NW_) that, like vcCNT, is 38% identical in amino acid sequence to hCNT3 (Hirschi *et al.*, 2017, *Nature* 10.1038/nature22057). The structures define the conformational transitions of the CNT elevator-type mechanism of transport, in which the permeant binding site moves a large distance across the cell membrane. In comparison with the corresponding inward-facing conformation, the outward-facing conformation of CNT_NW_ reveals a structure in which the scaffold domain is largely unchanged, while the transport domain moves ∼12 Å toward the extracellular side of the membrane, placing the nucleoside- (and Na^+^) binding sites above TM6 (equivalent to TM9 of hCNT3), and even more accessible to the extracellular medium than in the outward-facing repeat-swap model of hCNT3 presented in supplemental Fig. S4.
