# Supplementary material for: Substituted cysteine accessibility method (SCAM) analysis of the transport domain of human concentrative nucleoside transporter 3 (hCNT3) and other family members reveals features of structural and functional importance
Source: J Biol Chem. 2017 Apr 6;292(23):9505–22. doi: 10.1074/jbc.M116.743997 (PMC5465479; doi:10.1074/jbc.M116.743997)
Supplement: Supplemental Data [file supp_292_23_9505__index.html]

Substituted Cysteine Accessibility Method (SCAM) Analysis of the Transport Domain of Human Concentrative Nucleoside Transporter 3 (hCNT3) and Other Family Members Reveals Features of Structural and Functional Importance — Substituted cysteine accessibility method (SCAM) analysis of the transport domain of human concentrative nucleoside transporter 3 (hCNT3) and other family members reveals features of structural and functional importance — hCNT3 SCAM — Supplemental Data 

# Substituted cysteine accessibility method (SCAM) analysis of the transport domain of human concentrative nucleoside transporter 3 (hCNT3) and other family members reveals features of structural and functional importance

## Supplemental Data

- Supplemental Tables 1-7 and Supplemental Figures 1-4 (.docx, 2.1 MB) - Supplemental Tables 1 -7 and Supplemental Figures 1-4
