# Supplementary material for: Substituted cysteine accessibility method (SCAM) analysis of the transport domain of human concentrative nucleoside transporter 3 (hCNT3) and other family members reveals features of structural and functional importance
Source: J Biol Chem. 2017 Apr 6;292(23):9505–22. doi: 10.1074/jbc.M116.743997 (PMC5465479; doi:10.1074/jbc.M116.743997)
Supplement: Supplemental Data [file 10.1074_M116.743997_jbc.M116.743997-1.docx]

Supplemental Table 1. Na^+^- and H^+^-mediated uptake of uridine in *Xenopus* oocytes expressing hCNT3(C-) single cysteine mutants. Influx of 10 μM ^3^H-uridine was measured in either Na^+^-containing, H^+^-reduced or Na^+^-free, acidified media (100 mM NaCl, pH 8.5 or 100 mM ChCl, pH 5.5, respectively). Na^+^:H^+^ uptake ratios which were < 0.5 or > 2.5 are highlighted with an asterisk (*). Values are corrected for basal non-mediated uptake in control water-injected oocytes. Each value is the mean ± S.E.M. of 10-12 oocytes.

|  | Mediated Uridine Uptake  (pmol/oocyte.min^-1^) | |  |
| --- | --- | --- | --- |
| IH2 | Na^+^  (100 mM NaCl, pH 8.5) | H^+^  (100 mM ChCl, pH 5.5) | Na^+^:H^+^  Ratio |
|  |  |  |  |
| M308C(C-) | 0.9 ± 0.2 | 0.5 ± 0.1 | 1.8 |
| Q309C(C-) | 1.6 ± 0.3 | 1.7 ± 0.2 | 0.9 |
| W310C(C-) | 2.0 ± 0.4 | 1.4 ± 0.2 | 1.4 |
| I311C(C-) | 2.4 ± 0.2 | 1.6 ± 0.2 | 1.5 |
| I312C(C-) | 3.7 ± 0.3 | 2.7 ± 0.2 | 1.4 |
| R313C(C-) | 2.8 ± 0.4 | 1.7 ± 0.1 | 1.7 |
| K314C(C-) | 0.5 ± 0.1 | 0.4 ± 0.1 | 1.3 |
| V315C(C-) | 4.5 ± 0.4 | 2.8 ± 0.2 | 1.6 |
| G316C(C-) | 0.7 ± 0.1 | 0.6 ± 0.1 | 1.2 |
| W317C(C-) | 0.8 ± 0.1 | 1.0 ± 0.1 | 0.8 |
| I318C(C-) | 5.1 ± 0.4 | 3.1 ± 0.3 | 1.7 |
| M319C(C-) | 1.3 ± 0.2 | 2.5 ± 0.1 | 0.5 |
| L320C(C-) | 4.5 ± 0.4 | 2.8 ± 0.3 | 1.6 |
| V321C(C-) | 4.6 ± 0.4 | 3.9 ± 0.3 | 1.2 |
| T322C(C-) | 4.3 ± 0.4 | 2.1 ± 0.2 | 2.0 |
| T323C(C-) | 2.3 ± 0.3 | 2.2 ± 0.2 | 1.1 |
|  |  | |  |

|  | Mediated Uridine Uptake  (pmol/oocyte.min^-1^) | |  |
| --- | --- | --- | --- |
| IH2 and HP1a loop | Na^+^  (100 mM NaCl, pH 8.5) | H^+^  (100 mM ChCl, pH 5.5) | Na^+^:H^+^  Ratio |
|  |  |  |  |
| G324C(C-) | 2.1 ± 0.2 | 1.3 ± 0.1 | 1.6 |
| S325C(C-) | 3.0 ± 0.2 | 2.3 ± 0.1 | 1.3 |
| S326C(C-) | 0.7 ± 0.1 | 1.4 ± 0.1 | 0.5 |
|  |  | |  |

Table 1 continued

|  | Mediated Uridine Uptake  (pmol/oocyte.min^-1^) | |  |
| --- | --- | --- | --- |
| HP1a | Na^+^  (100 mM NaCl, pH 8.5) | H^+^  (100 mM ChCl, pH 5.5) | Na^+^:H^+^  Ratio |
|  |  |  |  |
| P327C(C-) | 1.8 ± 0.1 | 2.3 ± 0.3 | 0.8 |
| I328C(C-) | 3.4 ± 0.3 | 3.7 ± 0.2 | 0.9 |
| E329C(C-) | 1.7 ± 0.2 | 0.9 ± 0.2 | 1.9 |
| S330C(C-) | 1.6 ± 0.3 | 0.6 ± 0.1 | 2.7* |
| V331C(C-) | 2.0 ± 0.2 | 1.4 ± 0.1 | 1.5 |
| V332C(C-) | 3.8 ± 0.5 | 1.8 ± 0.3 | 2.1 |
| A333C(C-) | 1.9 ± 0.3 | 1.1 ± 0.2 | 1.8 |
| S334C(C-) | 0.8 ± 0.1 | 2.9 ± 0.2 | 0.3* |
| G335C(C-) | 3.5 ± 0.4 | 1.0 ± 0.2 | 3.5* |
| N336C(C-) | 1.3 ± 0.1 | 0.2 ± 0.1 | 6.5* |
| I337C(C-) | 0.7 ± 0.1 | 2.0 ± 0.1 | 0.4* |
| F338C(C-) | 1.2 ± 0.1 | 0.6 ± 0.1 | 2.0 |
|  |  |  |  |
|  |  |  |  |
|  |  |  |  |
|  | Mediated Uridine Uptake  (pmol/oocyte.min^-1^) | |  |
| HP1a and HP1b loop | Na^+^  (100 mM NaCl, pH 8.5) | H^+^  (100 mM ChCl, pH 5.5) | Na^+^:H^+^  Ratio |
|  |  |  |  |
| V339C(C-) | 4.7 ± 0.1 | 1.6 ± 0.1 | 2.9* |
| G340C(C-) | 2.1 ± 0.2 | 0.2 ± 0.1 | 10.2* |
| Q341C(C-) | 3.6 ± 0.2 | 0.8 ± 0.1 | 4.6* |
|  |  |  |  |
|  |  |  |  |
|  |  |  |  |

Table 1 continued

|  |  |  |  |
| --- | --- | --- | --- |
|  | Mediated Uridine Uptake  (pmol/oocyte.min^-1^) | |  |
| HP1b | Na^+^  (100 mM NaCl, pH 8.5) | H^+^  (100 mM ChCl, pH 5.5) | Na^+^:H^+^  Ratio |
|  |  |  |  |
| T342C(C-) | 4.0 ± 0.6 | 3.4 ± 0.2 | 1.2 |
| E343C(C-) | < 0.1 | < 0.1 | - |
| S344C(C-) | 4.7 ± 0.2 | 0.5 ± 0.1 | 9.4* |
| P345C(C-) | 5.0 ± 0.3 | 0.5 ± 0.1 | 9.2* |
| L346C(C-) | 6.2 ± 0.2 | 3.5 ± 0.3 | 1.8 |
| L347C(C-) | 3.9 ± 0.3 | 2.2 ± 0.1 | 1.8 |
| V348C(C-) | 4.3 ± 0.2 | 2.4 ± 0.2 | 1.8 |
| R349C(C-) | 4.9 ± 0.6 | 4.1 ± 0.6 | 1.2 |
| P350C(C-) | 5.5 ± 0.3 | 3.1 ± 0.1 | 1.8 |
|  |  | |  |

|  | Mediated Uridine Uptake  (pmol/oocyte.min^-1^) | |  |
| --- | --- | --- | --- |
| TM HP1b and TM7a Loop | Na^+^  (100 mM NaCl, pH 8.5) | H^+^  (100 mM ChCl, pH 5.5) | Na^+^:H^+^  Ratio |
|  |  |  |  |
| Y351C(C-) | 4.6 ± 0.2 | 3.7 ± 0.2 | 1.2 |
| L352C(C-) | 3.6 ± 0.5 | 0.8 ± 0.1 | 4.5* |
| P353C(C-) | 4.0 ± 0.4 | 3.5 ± 0.1 | 1.1 |
| Y354C(C-) | 5.2 ± 0.4 | 3.4 ± 0.2 | 1.5 |
| I355C(C-) | 5.4 ± 0.5 | 2.2 ± 0.2 | 2.5 |
|  |  |  |  |

Table 1 continued

|  | Mediated Uridine Uptake  (pmol/oocyte.min^-1^) | |  |
| --- | --- | --- | --- |
| TM7a | Na^+^  (100 mM NaCl, pH 8.5) | H^+^  (100 mM ChCl, pH 5.5) | Na^+^:H^+^  Ratio |
|  |  |  |  |
| T356C(C-) | 0.8 ± 0.1 | 0.6 ± 0.2 | 1.3 |
| K357C(C-) | 2.9 ± 0.5 | 2.9 ± 0.4 | 1.0 |
| S358C(C-) | 3.6 ± 0.4 | 2.1 ± 0.4 | 1.7 |
| E359C(C-) | 0.3 ± 0.1 | 0.2 ± 0.1 | 1.5 |
| L360C(C-) | 4.5 ± 0.4 | 1.8 ± 0.2 | 2.5 |
| H361C(C-) | 3.6 ± 0.3 | 1.5 ± 0.4 | 2.3 |
| A362C(C-) | 6.4 ± 0.3 | 4.1 ± 0.3 | 1.5 |
| I363C(C-) | 5.3 ± 0.2 | 2.6 ± 0.1 | 2.1 |
| M364C(C-) | 5.0 ± 0.4 | 2.6± 0.3 | 1.9 |
| T365C(C-) | 6.3 ± 0.9 | 4.3 ± 0.4 | 1.4 |
| A366C(C-) | 4.5 ± 0.3 | 4.6 ± 0.4 | 1.0 |
| G367C(C-) | 3.6 ± 0.3 | 1.5 ± 0.1 | 2.4 |
| F368C(C-) | 0.3 ± 0.1 | 0.6 ± 0.1 | 0.5 |
| S369C(C-) | 4.4 ± 0.6 | 1.5 ± 0.2 | 2.9* |
|  |  |  |  |

|  | Mediated Uridine Uptake  (pmol/oocyte.min^-1^) | |  |
| --- | --- | --- | --- |
| TM7a and TM7b loop | Na^+^  (100 mM NaCl, pH 8.5) | H^+^  (100 mM ChCl, pH 5.5) | Na^+^:H^+^  Ratio |
|  |  |  |  |
| T370C(C-) | 1.5 ± 0.1 | 7.1 ± 0.5 | 0.2* |
| I371C(C-) | 1.3 ± 0.1 | 0.7 ± 0.1 | 1.9 |
| A372C(C-) | 0.7 ± 0.1 | 0.6 ± 0.1 | 1.2 |
| G373C(C-) | 0.7 ± 0.1 | 0.5 ± 0.1 | 1.4 |
| S374C(C-) | 4.5 ± 0.4 | 2.7 ± 0.3 | 1.7 |
|  |  |  |  |

Table 1 continued

|  | Mediated Uridine Uptake  (pmol/oocyte.min^-1^) | |  |
| --- | --- | --- | --- |
| TM7b | Na^+^  (100 mM NaCl, pH 8.5) | H^+^  (100 mM ChCl, pH 5.5) | Na^+^:H^+^  Ratio |
|  |  |  |  |
| V375C(C-) | 1.6 ± 0.2 | 0.2 ± 0.1 | 8.0* |
| L376C(C-) | 1.9 ± 0.1 | 1.4 ± 0.1 | 1.4 |
| G377C(C-) | 1.9 ± 0.2 | 1.5 ± 0.2 | 1.3 |
| A378C(C-) | 4.0 ± 0.2 | 2.3 ± 0.2 | 1.7 |
| Y379C(C-) | < 0.1 | < 0.1 | - |
| I380C(C-) | 3.1 ± 0.2 | 2.1 ± 0.4 | 1.5 |
| S381C(C-) | 3.8 ± 1.0 | 2.4 ± 0.2 | 1.6 |
| F382C(C-) | 2.0 ± 0.2 | 1.1 ± 0.1 | 1.7 |
|  |  |  |  |

|  | Mediated Uridine Uptake  (pmol/oocyte.min^-1^) | |  |
| --- | --- | --- | --- |
| TM7b and TM8a Loop | Na^+^  (100 mM NaCl, pH 8.5) | H^+^  (100 mM ChCl, pH 5.5) | Na^+^:H^+^  Ratio |
|  |  |  |  |
| G383C(C-) | 3.0 ± 0.3 | 1.2 ± 0.1 | 2.5 |
| V384C(C-) | 0.9 ± 0.1 | 0.6 ± 0.1 | 1.4 |
| P385C(C-) | 1.4 ± 0.2 | 1.3 ± 0.1 | 1.2 |
|  |  |  |  |

Table 1 continued

|  | Mediated Uridine Uptake  (pmol/oocyte.min^-1^) | |  |
| --- | --- | --- | --- |
| TM8a | Na^+^  (100 mM NaCl, pH 8.5) | H^+^  (100 mM ChCl, pH 5.5) | Na^+^:H^+^  Ratio |
|  |  |  |  |
|  |  |  |  |
| S386C(C-) | 2.5 ± 0.2 | 2.1 ± 0.2 | 1.3 |
| S387C(C-) | 1.7 ± 0.1 | 2.3 ± 0.2 | 0.7 |
| L389C(C-) | 1.2 ± 0.1 | 0.2 ± 0.1 | 5.9* |
| L390C(C-) | 1.3 ± 0.2 | 1.6 ± 0.4 | 0.8 |
| T391C(C-) | 3.7 ± 0.2 | 4.5 ± 0.2 | 0.8 |
| A392C(C-) | 1.1 ± 0.2 | 0.7 ± 0.1 | 1.5 |
| S393C(C-) | 3.3 ± 0.4 | 1.5 ± 0.3 | 2.2 |
| V394C(C-) | 4.2 ± 0.3 | 1.7 ± 0.2 | 2.4 |
| M395C(C-) | 0.6 ± 0.1 | 1.6 ± 0.1 | 0.4* |
| S396C(C-) | 0.5 ± 0.1 | 0.3 ± 0.1 | 1.7 |
| A397C(C-) | 5.0 ± 0.4 | 3.3 ± 0.2 | 1.5 |
|  |  |  |  |

|  | Mediated Uridine Uptake  (pmol/oocyte.min^-1^) | |  |
| --- | --- | --- | --- |
| TM8a and TM8b loop | Na^+^  (100 mM NaCl, pH 8.5) | H^+^  (100 mM ChCl, pH 5.5) | Na^+^:H^+^  Ratio |
|  |  |  |  |
| P398C(C-) | 0.2 ± 0.1 | 0.2 ± 0.1 | 1.2 |
|  |  |  |  |

Table 1 continued

|  | Mediated Uridine Uptake  (pmol/oocyte.min^-1^) | |  |
| --- | --- | --- | --- |
| TM8b | Na^+^  (100 mM NaCl, pH 8.5) | H^+^  (100 mM ChCl, pH 5.5) | Na^+^:H^+^  Ratio |
|  |  |  |  |
| A399C(C-) | 4.4 ± 0.5 | 3.0 ± 0.2 | 1.5 |
| S400C(C-) | 2.7 ± 0.5 | 1.2 ± 0.1 | 2.3 |
| L401C(C-) | 1.8 ± 0.1 | 1.6 ± 0.3 | 1.2 |
| A402C(C-) | 3.7 ± 0.2 | 3.8 ± 0.1 | 1.0 |
| A403C(C-) | 3.8 ± 0.1 | 4.2 ± 0.2 | 0.9 |
| A404C(C-) | 2.3 ± 0.2 | 1.2 ± 0.2 | 1.9 |
| K405C(C-) | 2.6 ± 0.2 | 1.5 ± 0.1 | 1.7 |
| L406C(C-) | 3.0 ± 0.2 | 3.1 ± 0.2 | 1.0 |
| F407C(C-) | 2.7 ± 0.2 | 3.3 ± 0.3 | 0.8 |
| W408C(C-) | 1.4 ± 0.2 | 1.0 ± 0.2 | 1.4 |
|  |  |  |  |

Table 1 continued

|  | Mediated Uridine Uptake  (pmol/oocyte.min^-1^) | |  |
| --- | --- | --- | --- |
| TM8b and 9 Loop | Na^+^  (100 mM NaCl, pH 8.5) | H^+^  (100 mM ChCl, pH 5.5) | Na^+^:H^+^  Ratio |
|  |  |  |  |
| P409C(C-) | 3.1 ± 0.2 | 1.4 ± 0.1 | 2.2 |
| E410C(C-) | 1.3 ± 0.1 | 1.1 ± 0.1 | 1.3 |
| T411C(C-) | 1.6 ± 0.1 | 1.0 ± 0.1 | 1.6 |
| E412C(C-) | 1.3 ± 0.2 | 0.6 ± 0.1 | 2.4 |
| K413C(C-) | 0.8 ± 0.2 | 0.8 ± 0.2 | 1.0 |
| P414C(C-) | 0.9 ± 0.1 | 0.5 ± 0.1 | 1.7 |
| K415C(C-) | 1.4 ± 0.2 | 0.8 ± 0.1 | 1.7 |
| I416C(C-) | 1.4 ± 0.2 | 1.3 ± 0.2 | 1.1 |
| T417C(C-) | 1.9 ± 0.2 | 1.4 ± 0.2 | 1.4 |
| L418C(C-) | 1.7 ± 0.1 | 0.7 ± 0.1 | 2.5 |
| K419C(C-) | 1.5 ± 0.2 | 0.8 ± 0.1 | 1.9 |
| N420C(C-) | 1.5 ± 0.1 | 1.2 ± 0.1 | 1.3 |
| A421C(C-) | 0.6 ± 0.1 | 0.5 ± 0.1 | 1.2 |
| M422C(C-) | 0.2 ± 0.1 | 0.2 ± 0.1 | 1.0 |
| K423C(C-) | 4.0 ± 0.3 | 2.5 ± 0.2 | 1.6 |
| M424C(C-) | 1.7 ± 0.1 | 2.2 ± 0.1 | 0.8 |
| E425C(C-) | 2.3 ± 0.1 | 1.8 ± 0.1 | 1.3 |
| S426C(C-) | 2.8 ± 0.2 | 2.8 ± 0.1 | 1.0 |
| G427C(C-) | 3.5 ± 0.3 | 2.9 ± 0.2 | 1.2 |
| D428C(C-) | 3.4 ± 0.3 | 2.8 ± 0.1 | 1.2 |
| S429C(C-) | 3.3 ± 0.2 | 2.9 ± 0.3 | 1.2 |
| G430C(C-) | 2.9 ± 0.2 | 2.9 ± 0.1 | 1.0 |
| N431C(C-) | 1.4 ± 0.1 | 1.9 ± 0.2 | 0.7 |
|  |  |  |  |

Table 1 continued

|  | Mediated Uridine Uptake  (pmol/oocyte.min^-1^) | |  |
| --- | --- | --- | --- |
| TM9 | Na^+^  (100 mM NaCl, pH 8.5) | H^+^  (100 mM ChCl, pH 5.5) | Na^+^:H^+^  Ratio |
|  |  |  |  |
| L432C(C-) | 2.5 ± 0.3 | 2.0 ± 0.2 | 1.3 |
| L433C(C-) | 2.8 ± 0.2 | 1.8 ± 0.2 | 1.6 |
| E434C(C-) | 2.2 ± 0.2 | 1.4 ± 0.1 | 1.6 |
| A435C(C-) | 2.9 ± 0.2 | 2.8 ± 0.1 | 1.0 |
| A436C(C-) | 2.8 ± 0.2 | 2.3 ± 0.1 | 1.2 |
| T437C(C-) | 3.5 ± 0.2 | 2.9 ± 0.2 | 1.2 |
| Q438C(C-) | 2.9 ± 0.2 | 2.5 ± 0.1 | 1.2 |
| G439C(C-) | 4.1 ± 0.5 | 2.6 ± 0.3 | 1.6 |
| A440C(C-) | 2.2 ± 0.1 | 1.9 ± 0.1 | 1.1 |
| S441C(C-) | 0.5 ± 0.1 | 0.5 ± 0.1 | 1.0 |
| S442C(C-) | 0.7 ± 0.1 | 0.7 ± 0.1 | 1.0 |
| S443C(C-) | 0.7 ± 0.1 | 0.5 ± 0.1 | 1.4 |
| I444C(C-) | 0.6 ± 0.1 | 0.7 ± 0.1 | 0.9 |
| S445C(C-) | 0.7 ± 0.1 | 0.7 ± 0.1 | 1.0 |
| L446C(C-) | 1.8 ± 0.2 | 2.5 ± 0.2 | 0.7 |
| V447C(C-) | 2.7 ± 0.3 | 4.1 ± 0.2 | 0.7 |
| A448C(C-) | 2.9 ± 0.1 | 3.0 ± 0.2 | 0.9 |
| N449C(C-) | 2.9 ± 0.4 | 3.7 ± 0.3 | 0.8 |
| I450C(C-) | 1.6 ± 0.1 | 2.7 ± 0.2 | 0.6 |
| A451C(C-) | 2.5 ± 0.1 | 4.0 ± 0.4 | 0.6 |
| V452C(C-) | 2.3 ± 0.3 | 4.1 ± 0.2 | 0.6 |
| N453C(C-) | 3.2 ± 0.1 | 3.7 ± 0.2 | 0.9 |
| L454C(C-) | 1.1 ± 0.1 | 0.6 ± 0.1 | 1.7 |
| I455C(C-) | 3.2 ± 0.1 | 2.3 ± 0.1 | 1.4 |
| A456C(C-) | 1.7 ± 0.1 | 1.4 ± 0.1 | 1.2 |
| F457C(C-) | 1.4 ± 0.1 | 1.1 ± 0.1 | 1.3 |
| L458C(C-) | 2.8 ± 0.2 | 3.0 ± 0.2 | 1.0 |
| A459C(C-) | 4.5 ± 0.1 | 2.9 ± 0.3 | 1.6 |
| L460C(C-) | 3.9 ± 0.1 | 3.7 ± 0.3 | 1.0 |
| L461C(C-) | 0.9 ± 0.1 | 2.5 ± 0.3 | 0.4* |
| S462C(C-) | 3.4 ± 0.2 | 3.1 ± 0.3 | 1.1 |
| F463C(C-) | 3.1 ± 0.2 | 2.6 ± 0.3 | 1.2 |
| M464C(C-) | 2.2 ± 0.2 | 2.7 ± 0.3 | 0.8 |
|  |  |  |  |
|  |  |  |  |
| N465C(C-) | 0.7 ± 0.1 | 0.5 ± 0.1 | 1.5 |
| S466C(C-) | 3.1 ± 0.2 | 2.7 ± 0.2 | 1.1 |
| A467C(C-) | 2.0 ± 0.1 | 2.1 ± 0.1 | 0.9 |
| L468C(C-) | 0.5 ± 0.1 | 0.6 ± 0.1 | 0.8 |
| S469C(C-) | 2.7 ± 0.2 | 1.7 ± 0.1 | 1.6 |
| W470C(C-) | 0.5 ± 0.1 | 0.5 ± 0.1 | 1.0 |
| F471C(C-) | 0.5 ± 0.1 | 0.6 ± 0.1 | 0.9 |
| G472C(C-) | 16 ± 2.1 | 14 ± 0.8 | 1.1 |
| N473C(C-) | 0.8 ± 0.1 | 0.5 ± 0.1 | 1.7 |
| M474C(C-) | 0.6 ± 0.1 | 0.4 ± 0.1 | 1.6 |
| F475C(C-) | 0.7 ± 0.1 | 0.5 ± 0.1 | 1.6 |
|  |  |  |  |

Table 1 continued

|  | Mediated Uridine Uptake  (pmol/oocyte.min^-1^) | |  |
| --- | --- | --- | --- |
| Loop between TM9 and IH3 | Na^+^  (100 mM NaCl, pH 8.5) | H^+^  (100 mM ChCl, pH 5.5) | Na^+^:H^+^  Ratio |
|  |  |  |  |
| D476C(C-) | 0.9 ± 0.1 | 0.5 ± 0.1 | 1.8 |
| Y477C(C-) | 0.7 ± 0.1 | 0.5 ± 0.1 | 1.4 |
| P478C(C-) | 0.2 ± 0.1 | 0.2 ± 0.1 | 1.0 |
| Q479C(C-) | 0.4 ± 0.1 | 0.3 ± 0.1 | 1.3 |
|  |  |  |  |

**Supplemental Table 2. Uptake of uridine in *Xenopus* oocytes expressing hCNT1 single cysteine mutants.** Influx of 10 μM ^3^H-uridine was measured in Na^+^-containing medium (100 mM NaCl, pH 8.5). Values are corrected for basal non-mediated uptake in control water-injected oocytes. Each value is the mean ± S.E.M. of 10-12 oocytes.

|  | | | | |
| --- | --- | --- | --- | --- |
| Mediated Uridine Uptake  (pmol/oocyte.min^-1^)  (100 mM NaCl, pH 8.5) | | | | |
|  |  |  |  |  |
| HP1a and HP1b |  |  | TM7a |  |
|  |  |  |  |  |
| T307C | 5.5 ± 0.4 |  | V339C | 5.5 ± 0.2 |
| E308C | 0.7 ± 0.1 |  | H340C | 6.0 ± 0.4 |
| T309C | 4.7 ± 0.4 |  | V341C | 4.0 ± 0.3 |
| L310C | 4.8 ± 0.3 |  | V342C | 5.9 ± 0.3 |
| S311C | 5.0 ± 0.4 |  | M343C | 4.5 ± 0.4 |
| V312C | 3.7 ± 0.2 |  | T344C | 4.8 ± 0.3 |
| A313C | 3.3 ± 0.2 |  | G345C | 3.3 ± 0.2 |
| G314C | 4.6 ± 0.4 |  | G346C | 0.5 ± 0.1 |
| N315C | 1.0 ± 0.1 |  | Y347C | 1.0 ± 0.2 |
| I316C | 2.9 ± 0.3 |  | A348C | 2.3 ± 0.4 |
| F317C | 4.9 ± 0.3 |  | T349C | 0.2 ± 0.1 |
| V318C | 4.3 ± 0.4 |  | I350C | 1.9 ± 0.2 |
| S319C | 0.9 ± 0.1 |  | A351C | 1.0 ± 0.1 |
| Q320C | 2.6 ± 0.3 |  | G352C | 3.5 ± 0.2 |
| T321C | 0.7 ± 0.1 |  | S353C | 2.2 ± 0.2 |
| E322C | 0.3 ± 0.1 |  | L354C | 1.7 ± 0.2 |
| A323C | 3.7 ± 0.3 |  | L355C | 1.8 ± 0.2 |
| P324C | 1.5 ± 0.1 |  | G356C | 0.9 ± 0.2 |
| L325C | 3.5 ± 0.2 |  | A357C | 3.2 ± 0.2 |
| L326C | 2.5 ± 0.2 |  | Y358C | 1.0 ± 0.1 |
| I327C | 3.9 ± 0.3 |  | I359C | 4.6 ± 0.2 |
| TM7b and TM8a Loop |  |  |  |  |
| I363C | 2.3 ± 0.3 |  | hCNT1 | 8.9 ± 0.6 |

**Supplemental Table 3. Effects of PCMBS on uridine uptake in *Xenopus* oocytes expressing hCNT1 single cysteine mutants.** Influx of 10 μM ^3^H-uridine was measured in Na^+^-containing media (100 mM NaCl, pH 8.5) following 10 min incubation on ice in the absence or presence of 200 μM PCMBS or 200 μM PCMBS + 20 mM uridine in media of the same composition used to determine uptake. Values are corrected for basal non-mediated uptake in control water-injected oocytes and are presented as a percentage of mediated uridine influx in the absence of inhibitor for each individual mutant. Each value is the mean ± S.E.M. of 10-12 oocytes. The symbol ^*^ indicates substrate protection.

|  |  | Na^+^  (100 mM NaCl, pH 8.5) | |
| --- | --- | --- | --- |
| TM |  | + PCMBS^a^  (%) | + PCMBS  + uridine  (%) |
|  |  |  |  |
| HP1a and HP1b loop | S319C | 36 ± 3 | 50 ± 4 |
| HP1a and HP1b loop | Q320C | 47 ± 5 | ^*^89 ± 7 |
|  |  |  |  |
| HP1b | T321C | 31 ± 2 | ^*^96 ± 7 |
| HP1b | E322C | 21 ± 1 | ^*^106 ± 9 |
|  |  |  |  |
| TM7a and TM7b loop | I350C | 8 ± 1 | ^*^96 ± 7 |
| TM7a and TM7b loop | A351C | 46 ± 2 | 57 ± 4 |
| TM7a and TM7b loop | G352C | 15 ± 2 | ^*^88 ± 7 |
| TM7a and TM7b loop | S353C | 42 ± 3 | ^*^80 ± 4 |
|  |  |  |  |
| TM7b | L354C | 31 ± 3 | ^*^64 ± 3 |
| TM7b | L355C | 53 ± 6 | 51 ± 6 |
| TM7b | A357C | 58 ± 3 | 62 ± 4 |
| TM7b | Y358C | 27 ± 3 | 28 ± 3 |
|  |  |  |  |
| TM7b and TM8a Loop  Control | I363C  hCNT1 | 43 ± 2  108 ± 7 | 44 ± 4  88 ± 3 |

^a^, mediated uridine influx in the absence of inhibitor is given in

pmol/oocytes.min^-1^ in supplemental Table 2 for each of the individual mutants.

**Supplemental Table 4. Effects of** Na^+^ **on PCMBS inhibition of hCNT1 single cysteine mutants.** Influx of 10 μM ^3^H-uridine was measured in Na^+^-containing medium (100 mM NaCl, pH 8.5) following 10 min incubation on ice in the absence or presence of PCMBS in either Na^+^-free or Na^+^-containing medium (100 mM ChCl, pH 8.5 and 100 mM NaCl, pH 8.5, respectively). Values are corrected for basal non-mediated uptake in control water-injected oocytes and are normalized to the respective influx of uridine in the absence of inhibitor. Each value is the mean ± S.E.M. of 10-12 oocytes.

|  | Uptake (%)  (100 mM NaCl, pH 7.5) | | |
| --- | --- | --- | --- |
|  |  | PCMBS in Na^+^-free medium | PCMBS in Na^+^-containing medium |
|  |  |  |  |
| HP1a and HP1b loop | S319C | 22 ± 1 | 25 ± 4 |
| HP1a and HP1b loop | Q320C | 53 ± 5 | 46 ± 7 |
|  |  |  |  |
| HP1b | T321C | 10 ± 2 | 12 ± 4 |
| HP1b | E322C | 69 ± 6 | 34 ± 3 |
|  |  |  |  |
| TM7a and TM7b loop | I350C | 5 ± 1 | 7 ± 1 |
| TM7a and TM7b loop | A351C | 28 ± 3 | 40 ± 4 |
| TM7a and TM7b loop | G352C | 4 ± 0.4 | 7 ± 1 |
| TM7a and TM7b loop | S353C | 39 ± 3 | 38 ± 3 |
|  |  |  |  |
| TM7b | L354C | 20 ± 2 | 22 ± 1 |
| TM7b | L355C | 32 ± 2 | 26 ± 3 |
| TM7b | A357C | 52 ± 4 | 59 ± 4 |
| TM7b | Y358C | 6 ± 1 | 13 ± 2 |
|  |  |  |  |

**Supplemental Table 5: Uptake of uridine in *Xenopus* oocytes expressing NupC(C-) single cysteine mutants.** Influx of 1 μM ^3^H-uridine was measured in Na^+^-reduced, acidified media (100 mM ChCl, pH 5.5). Values are corrected for basal non-mediated uptake in control water-injected oocytes. Each value is the mean ± S.E.M. of 10-12 oocytes.

| Mediated Uridine Uptake  (pmol/oocyte.10 min^-1^)  H^+^  (100 mM ChCl, pH 5.5) | | | | |
| --- | --- | --- | --- | --- |
| HP1a and HP1b |  |  | TM4a and TM4b |  |
|  |  |  |  |  |
| L134C(C-) | 0.18 ± 0.02 |  | M166C(C-) | < 0.05 |
| E135C(C-) | < 0.05 |  | Y167C(C-) | 0.10 ± 0.01 |
| S136C(C-) | 0.50 ± 0.09 |  | T168C(C-) | 0.06 ± 0.01 |
| F137C(C-) | < 0.05 |  | M169C(C-) | 0.05 ± 0.01 |
| N138C(C-) | 0.14 ± 0.02 |  | A170C(C-) | 0.20 ± 0.04 |
| A139C(C-) | 0.11 ± 0.02 |  | A171C(C-) | 0.15 ± 0.02 |
| V140C(C-) | 0.49 ± 0.04 |  | T172C(C-) | < 0.05 |
| S141C(C-) | 0.39 ± 0.04 |  | A173C(C-) | 0.18 ± 0.02 |
| S142C(C-) | 0.47 ± 0.06 |  | M174C(C-) | 0.11 ± 0.01 |
| L143C(C-) | 0.77 ± 0.08 |  | S175C(C-) | < 0.05 |
| I144C(C-) | 0.18 ± 0.03 |  | T176C(C-) | < 0.05 |
| L145C(C-) | 0.48 ± 0.05 |  | V177C(C-) | 0.10 ± 0.01 |
| G146C(C-) | < 0.05 |  | S178C(C-) | 0.19 ± 0.02 |
| Q147C(C-) | 0.15 ± 0.02 |  | M179C(C-) | < 0.05 |
| S148C(C-) | 0.67 ± 0.06 |  | S180C(C-) | 0.15 ± 0.02 |
| E149C(C-) | 0.10 ± 0.01 |  | I181C(C-) | 0.56 ± 0.06 |
| N150C(C-) | 1.1 ± 0.1 |  | V182C(C-) | 0.05 ± 0.01 |
| F151C(C-) | < 0.05 |  | G183C(C-) | < 0.05 |
| I152C(C-) | < 0.05 |  | A184C(C-) | 0.08 ± 0.02 |
| A153C(C-) | 0.79 ± 0.10 |  | Y185C(C-) | 0.07 ± 0.01 |
| Y154C(C-) | 0.11 ± 0.03 |  | M186C(C-) | < 0.05 |
|  |  |  | L190C(C-) | 0.26 ± 0.02 |
| NupC(C-) | 0.64 ± 0.04 |  |  |  |
|  |  |  |  |  |

**Supplemental Table 6: Effects of PCMBS on uridine uptake in *Xenopus* oocytes expressing NupC(C-) single cysteine mutants.** Influx of 1 μM ^3^H-uridine was measured in Na^+^-reduced, acidified media (100 mM ChCl, pH 5.5) following 10 min incubation on ice in the absence or presence of 200 μM PCMBS or 200 μM PCMBS + 20 mM uridine in medium of the same composition used to determine uptake. Values are corrected for basal non-mediated uptake in control water-injected oocytes and are presented as a percentage of mediated uridine influx in the absence of inhibitor for each individual mutant. Each value is the mean ± S.E.M. of 10-12 oocytes. The symbol ^*^ indicates substrate protection.

|  |  | Uptake (%)  (100 mM ChCl, pH 5.5) | |
| --- | --- | --- | --- |
| TM |  | + PCMBS^a^ | + PCMBS  + uridine |
|  |  |  |  |
| HP1a | S142C (C-) | 56 ± 4 | 47 ± 4 |
|  |  |  |  |
| TM4b | S180C (C-) | 48 ± 9 | 45 ± 10 |
|  | I181C (C-) | 47 ± 6 | ^*^95 ± 14 |
|  | A184C (C-) | 43 ± 2 | 45 ± 4 |
| Control | Y185C (C-)  NupC(C-) | 34 ± 10  96 ± 1 | 40 ± 11  - |

^a^, mediated uridine influx in the absence of inhibitor is given in pmol/oocytes.10 min^-1^ in supplemental Table 5 for each of the individual mutants.

**Supplemental Table 7: Effects of H^+^ on PCMBS inhibition of NupC(C-) single cysteine mutants.** Influx of 1 μM ^3^H-uridine was measured in Na^+^-reduced, acidified media (100 mM ChCl, pH 5.5; 10 min; 20 ºC) following 10 min incubation on ice in the absence or presence of PCMBS in either Na^+^-reduced, H^+^-reduced or Na^+^-reduced, acidified media medium (100 mM ChCl pH 8.5, 100 mM ChCl, pH 5.5, respectively). Values are corrected for basal non-mediated uptake in control water-injected oocytes and are normalized to the respective influx of uridine in the absence of inhibitor. Each value is the mean ± S.E.M. of 10-12 oocytes.

|  | | Uptake (%)  (100 mM ChCl, pH 5.5) | | |
| --- | --- | --- | --- | --- |
|  |  | | + PCMBS in H^+^-reduced medium | + PCMBS in acidified medium |
|  |  | |  |  |
| HP1a | S142C(C-) | | 39 ± 21 | 29 ± 7 |
|  |  | |  |  |
|  |  | |  |  |
| TM4b | S180C(C-) | | 53 ± 4 | 57 ± 5 |
| TM4b | I181C(C-) | | 50 ± 7 | 38 ± 7 |
| TM4b | A184C(C-) | | 11 ± 5 | 2 ± 3 |
| TM4b | Y185C(C-) | | 30 ± 6 | 5 ± 7 |

**Supplemental Figure 1: Topology of hCNT1**. Schematic hCNT1 topology. The position of endogenous cysteine residues are indicated as *black* residues and putative glycosylation sites are highlighted with a *star* symbol. Residues studied by SCAM analysis are depicted in the inset. Some constructs were not available and are denoted by a – symbol.

**Supplemental Figure 2: Topology of NupC**. Schematic NUPC topology. The position of endogenous cysteine residue is indicated as *black* residues Residues studied by SCAM analysis are depicted in the inset. Some constructs were not available and are denoted by a – symbol.

**(A)**

**(B)**

**Supplemental Figure 3**: PCMBS Inhibition of wild-type hCNT3: PCMBS inhibition time course and uridine protection. Influx of 10µM ^3^H-uridine in both Na^+^ -containing, H^+^-reduced and Na^+^-free, acidified media (A and B, respectively) was measured after timed exposure of hCNT3-producing oocytes to 200µM PCMBS (solid triangle) or 200µM PCMBS + 20 mM uridine (open circle) in media of the same composition used to determine uptake. Values are corrected for basal non-mediated uptake in control water-injected oocytes. Each value is the mean ± S.E.M. of 10-12 oocytes.


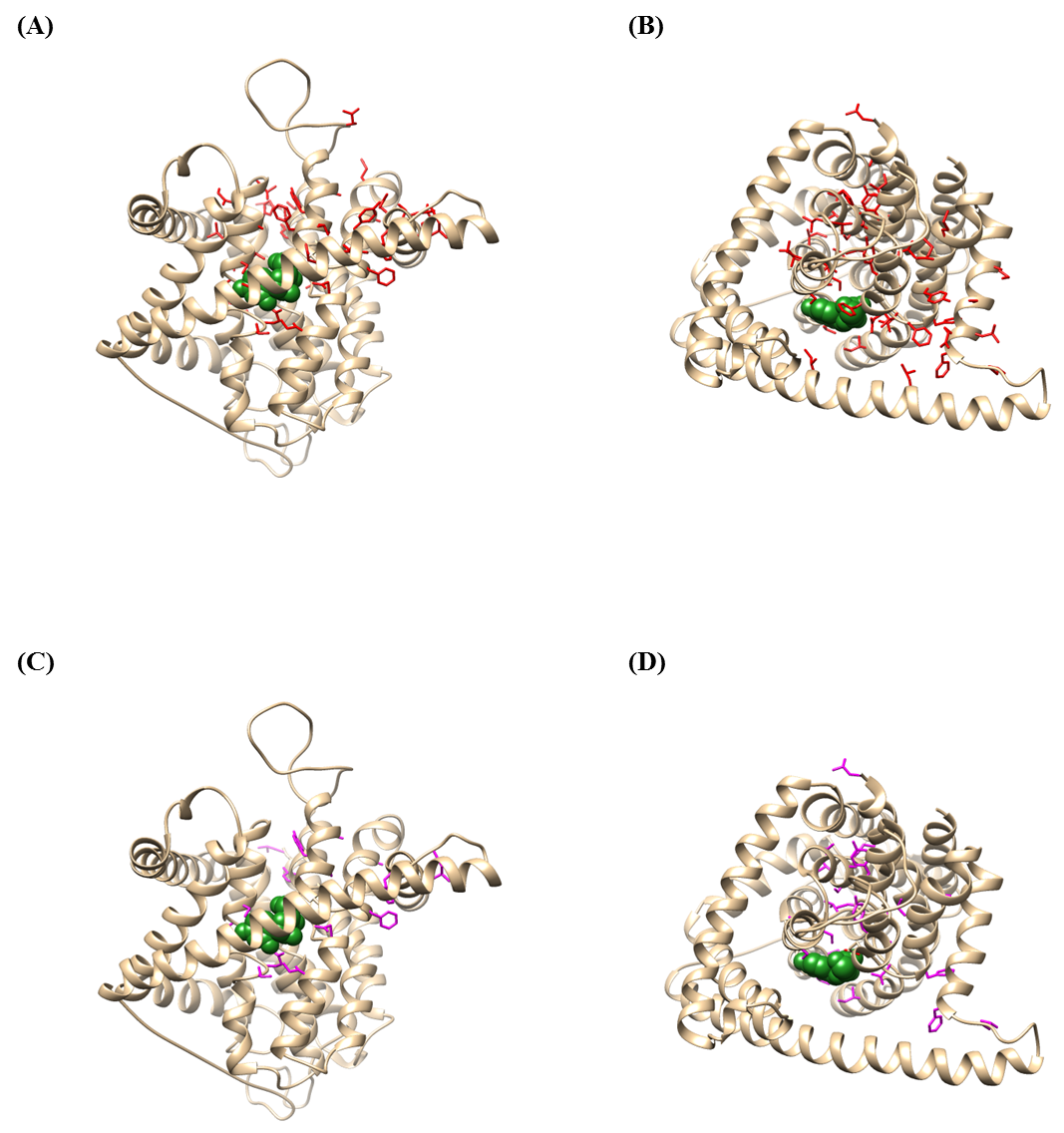


**Supplemental Figure 4**: Homology model of hCNT3. Cartoon representation of the outward-facing conformation of hCNT3 3D modelled upon the repeat-swap structural model of the bacterial nucleoside transporter vcCNT (PMDB identifier PM0080188) (50) using the program SWISS-MODEL (56). Molecular graphics and analyses were performed with the UCSF Chimera package (57). (A and C) Models of hCNT3 viewed parallel to the membrane. (B and D) Models of hCNT3 viewed from the extracellular surface of the membrane. Side chains of PCMBS-sensitive residues in A and B are shown in *red*. Side chains of PCMBS-sensitive and uridine-protected residues in C and D are shown in *purple*. Bound molecule of uridine is shown in space filling representation (*green*).
